# Mosquito Saliva Increases Endothelial Permeability in the Skin, Immune Cell Migration, and Dengue Pathogenesis during Antibody-Dependent Enhancement

**DOI:** 10.1371/journal.ppat.1005676

**Published:** 2016-06-16

**Authors:** Michael A. Schmid, Dustin R. Glasner, Sanjana Shah, Daniela Michlmayr, Laura D. Kramer, Eva Harris

**Affiliations:** 1 Division of Infectious Diseases and Vaccinology, School of Public Health, University of California, Berkeley, Berkeley, California, United States of America; 2 Wadsworth Center, New York State Department of Health, Albany, New York, United States of America; University of North Carolina at Chapel Hill, UNITED STATES

## Abstract

Dengue remains the most prevalent arthropod-borne viral disease in humans. While probing for blood vessels, *Aedes aegypti* and *Ae*. *albopictus* mosquitoes transmit the four serotypes of dengue virus (DENV1-4) by injecting virus-containing saliva into the skin. Even though arthropod saliva is known to facilitate transmission and modulate host responses to other pathogens, the full impact of mosquito saliva on dengue pathogenesis is still not well understood. Inoculating mice lacking the interferon-α/β receptor intradermally with DENV revealed that mosquito salivary gland extract (SGE) exacerbates dengue pathogenesis specifically in the presence of enhancing serotype-cross-reactive antibodies—when individuals already carry an increased risk for severe disease. We further establish that SGE increases viral titers in the skin, boosts antibody-enhanced DENV infection of dendritic cells and macrophages in the dermis, and amplifies dendritic cell migration to skin-draining lymph nodes. We demonstrate that SGE directly disrupts endothelial barrier function *in vitro* and induces endothelial permeability *in vivo* in the skin. Finally, we show that surgically removing the site of DENV transmission in the skin after 4 hours rescued mice from disease in the absence of SGE, but no longer prevented lethal antibody-enhanced disease when SGE was present. These results indicate that SGE accelerates the dynamics of dengue pathogenesis after virus transmission in the skin and induces severe antibody-enhanced disease systemically. Our study reveals novel aspects of dengue pathogenesis and suggests that animal models of dengue and pre-clinical testing of dengue vaccines should consider mosquito-derived factors as well as enhancing antibodies.

## Author Summary

Mosquitoes inject saliva into the skin while probing for blood vessels. Saliva facilitates blood feeding and can contain pathogens when the mosquito is infected. In tropical regions, *Aedes* mosquitoes transmit the four serotypes of dengue virus (DENV1-4) and infect almost 400 million humans every year. DENV causes severe disease especially in people who have already been exposed to a different serotype. During antibody-dependent enhancement, antibodies that were generated during the first infection bind, but do not neutralize, DENV, and instead enhance infection of immune cells. We injected mouse ears with DENV alone or with extracts from mosquito salivary glands to study the impact on disease. We found that saliva induced severe disease and death only during antibody-enhanced infection. Saliva increased DENV infection in the dermis, immune cell migration to skin and lymph nodes, and permeability of endothelial cells that line blood vessels. Removing the site of DENV inoculation in the skin rescued mice from severe disease, but this protective effect was lost when saliva was present. Our study reveals that mosquito saliva affects dendritic cell migration, increases endothelial permeability, and augments dengue disease severity. Mosquito saliva and enhancing antibodies thus need to be considered when developing vaccines and drugs against dengue.

## Introduction

During the transmission of arthropod-borne diseases, blood-feeding insects deposit pathogen-containing saliva into the skin [1]. How factors in arthropod saliva impact pathogen replication, host responses, and disease outcome is an area of active research. The four serotypes of dengue virus (DENV1-4) cause the most prevalent mosquito-borne viral disease, with up to 390 million infections and 96 million cases of dengue annually [2]. The first (1°) DENV infection usually causes dengue fever or subclinical disease, and individuals generate memory immune responses that protect against infection with the same DENV serotype. In contrast, sequential infection with a different DENV serotype can induce severe and potentially fatal disease [3]. Severe dengue is characterized by high fever, low platelet count, hemorrhagic manifestations, systemic vascular leak, and/or circulatory shock [4]. The memory immune response generated during a previous DENV infection can exacerbate a subsequent infection with a different serotype via a phenomenon known as antibody-dependent enhancement (ADE) and/or a cytokine storm produced by cross-reactive T cells [5,6]. During ADE, antibodies that were generated during the 1° infection and cross-react with the next infecting DENV serotype do not neutralize but instead enhance infection of Fc*γ* receptor-bearing cells, such as macrophages (MΦs) and monocytes [7–10]. Whereas monocytes circulate in the blood and enter inflamed tissues, MΦs and classical dendritic cells (cDCs) reside in steady-state tissues. After systemic dissemination, DENV infects monocytes in the blood [11], as well as MΦs and DCs in lymph nodes (LNs), spleen, intestine, and liver [12–16].

While probing for blood vessels in the skin, arthropods induce a host response that includes narrowing of blood vessels, blood clotting, and inflammation. To allow efficient blood feeding, arthropods inject saliva that contains various molecules that counteract this response [1]. Pathogens have co-evolved within their vector to optimize transmission [17]. *Plasmodium* [18,19] and *Leishmania* parasites [20,21] efficiently establish infections experimentally only when transmitted with vector saliva, and mosquito saliva enhances West Nile virus (WNV) infection [22,23]. Female *Aedes aegypti* and *Ae*. *albopictus* mosquitoes transmit DENV, Zika virus, yellow fever virus, and Chikungunya virus and are thus important vectors of disease. Nonetheless, the role of mosquito saliva in dengue pathogenesis is not well understood. *Ae*. *aegypti* saliva increased DENV serum viremia in mice lacking interferon (IFN) regulatory factors 3 and 7 (IRF3/7) [24] and prolonged dengue viremia in “humanized” mice xeno-transplanted with human hematopoietic stem and progenitor cells [25]. In contrast, Ader *et al*. found that mosquito saliva inhibits DENV infection of human monocyte-derived DCs (moDCs) *in vitro* [26]. Even though dermal cDCs and MΦs are the initial targets for DENV replication [27,28], the impact of mosquito saliva on immune cells in DENV-infected skin has not been studied.

Here we examined the impact of *Ae*. *aegypti* salivary gland extract (SGE) on dengue pathogenesis, the host response, and DENV infection in the skin. We used mice lacking the IFN-α/β receptor (*Ifnar* ^–/–^) that are susceptible to intradermal (i.d.) DENV infection [28] and display key features of human disease, such as lethal vascular leak [29–31]. In infected human cells, DENV proteins block IFN-α/β signaling [32–34]. In contrast, wild-type mice do not support DENV replication [35] because DENV proteins cannot block IFN signaling in mice [36]. While DENV inoculation of naïve *Ifnar* ^–/–^mice mimics 1° infection conditions, inoculation of mice previously injected with sub-neutralizing levels of DENV-specific antibodies models ADE [14,15,31]. We needle-inoculated *Ifnar* ^–/–^mice i.d. in the presence or absence of enhancing antibodies with DENV alone or DENV mixed with SGE from female *Ae*. *aegypti* mosquitoes—a model to examine the role of vector-derived factors in pathogen transmission that is more broadly accessible and easier to standardize than the use of live mosquitoes [37–40].

We establish here that only the combined presence of SGE and enhancing antibodies augments dengue pathogenesis, DENV infection of DCs and MΦs in the dermis, and immune responses in skin-draining LNs. In addition, we show that SGE increases endothelial permeability *in vitro* and induces vascular leak in the skin. Finally, removing the site of DENV infection in the ear after 4 hours (h) rescued mice from severe disease in the absence of SGE, but this rescue was lost when SGE was present. Mosquito-derived factors thus augment antibody-enhanced dengue pathogenesis in the skin and beyond.

## Results

### Mosquito SGE exacerbates dengue pathogenesis during ADE

**Fig 1:**
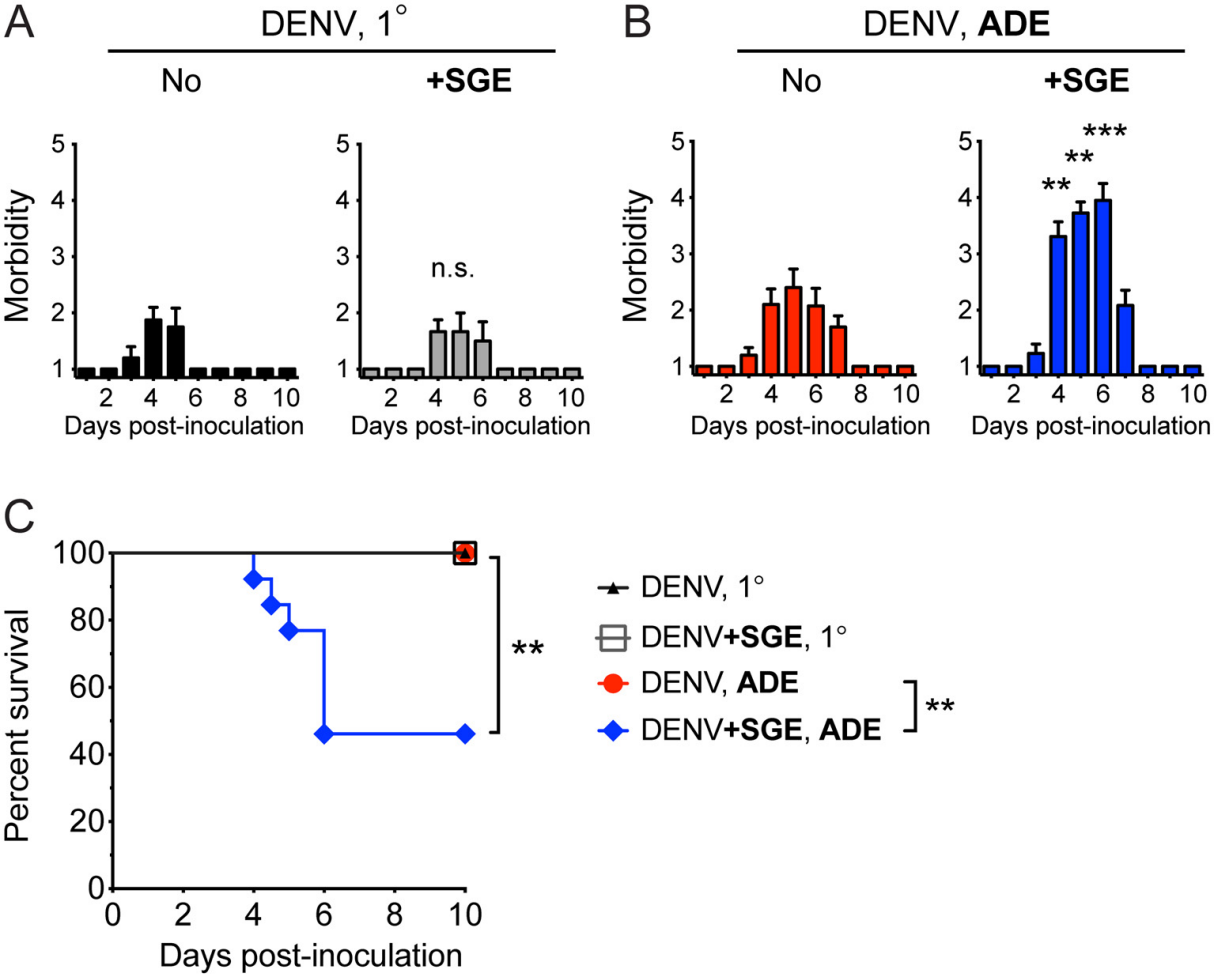
Mosquito SGE induces lethal antibody-enhanced dengue disease. *Ifnar* ^–/–^mice were inoculated i.d. with 10^5^ PFU DENV in the absence (A) or presence (B) of enhancing antibodies. DENV was inoculated alone (No) or after mixing with *Ae*. *aegypti* SGE (+SGE). (A-B) Bar graphs show mean morbidity ± standard error of mean (SEM) of mice on a scale from 1 = healthy to 5 = moribund (for details, see Material and Methods). (C) Kaplan-Meier curves showing survival of mice. Data were pooled from three experiments for 1° infection conditions n = 8 (No) or n = 6 (+SGE) and four experiments for ADE n = 10 (No) or n = 13 (+SGE). Statistically significant differences between the presence and absence of SGE are marked as ** for p<0.01, *** for p<0.001, or not significant (n.s. for p>0.05).

To determine the role of mosquito-derived factors on dengue pathogenesis, we inoculated *Ifnar* ^–/–^mice i.d. with 10^5^ PFU of the mouse-adapted virulent DENV2 strain D220 [31] (hereafter termed “DENV”) in PBS or mixed with SGE (equivalent to one salivary gland of a female *Ae*. *aegypti* mosquito). SGE had no effect on dengue pathogenesis in the absence of enhancing antibodies (1° infection), which induced mild or no disease (Fig 1A). In contrast, the presence of SGE significantly increased morbidity (Fig 1B) and induced lethal disease in 54% of the mice (Fig 1C) under antibody-enhanced conditions (ADE). The presence of enhancing antibodies mildly increased morbidity compared to 1° infection in the absence of SGE only on day 7 (p<0.05) but significantly increased morbidity on days 4, 5, 6 (p<0.001) and 7 (p<0.05) in the presence of SGE (Fig 1A and 1B). Severe dengue disease resulting from 10^5^ PFU DENV is thus dependent upon the combined presence of mosquito-derived factors in SGE and enhancing antibodies.

Due to the mild morbidity under 1° conditions that were observed after inoculation of 10^5^ PFU, we further tested the effect of SGE at a 10-fold higher DENV dose of 10^6^ PFU. Unexpectedly, SGE diminished disease severity under 1° conditions and did not further augment pathogenesis during antibody-enhanced DENV infection at 10^6^ PFU (S1 Fig), possibly because ADE of 10^6^ PFU already causes lethal disease. Because *Culex* mosquitoes transmit a median dose of 10^5^ PFU of the related Flavivirus, WNV [41], and a higher virus dose might override the enhancing effect of SGE on dengue pathogenesis, we subsequently used 10^5^ PFU DENV. In endemic areas, exposure to non-infected mosquitoes regularly occurs. In order to test whether pre-existing immunity to SGE would alter the effect of SGE on dengue pathogenesis, we inoculated *Ifnar* ^–/–^mice three times i.d. with SGE alone (at ≥2 weeks intervals). Subsequent i.d. antibody-enhanced DENV infection in the presence of SGE augmented pathogenesis similarly to that observed in SGE-naïve mice (S1 Fig).

### SGE boosts infection of dermal dendritic cells and macrophages

**Fig 2:**
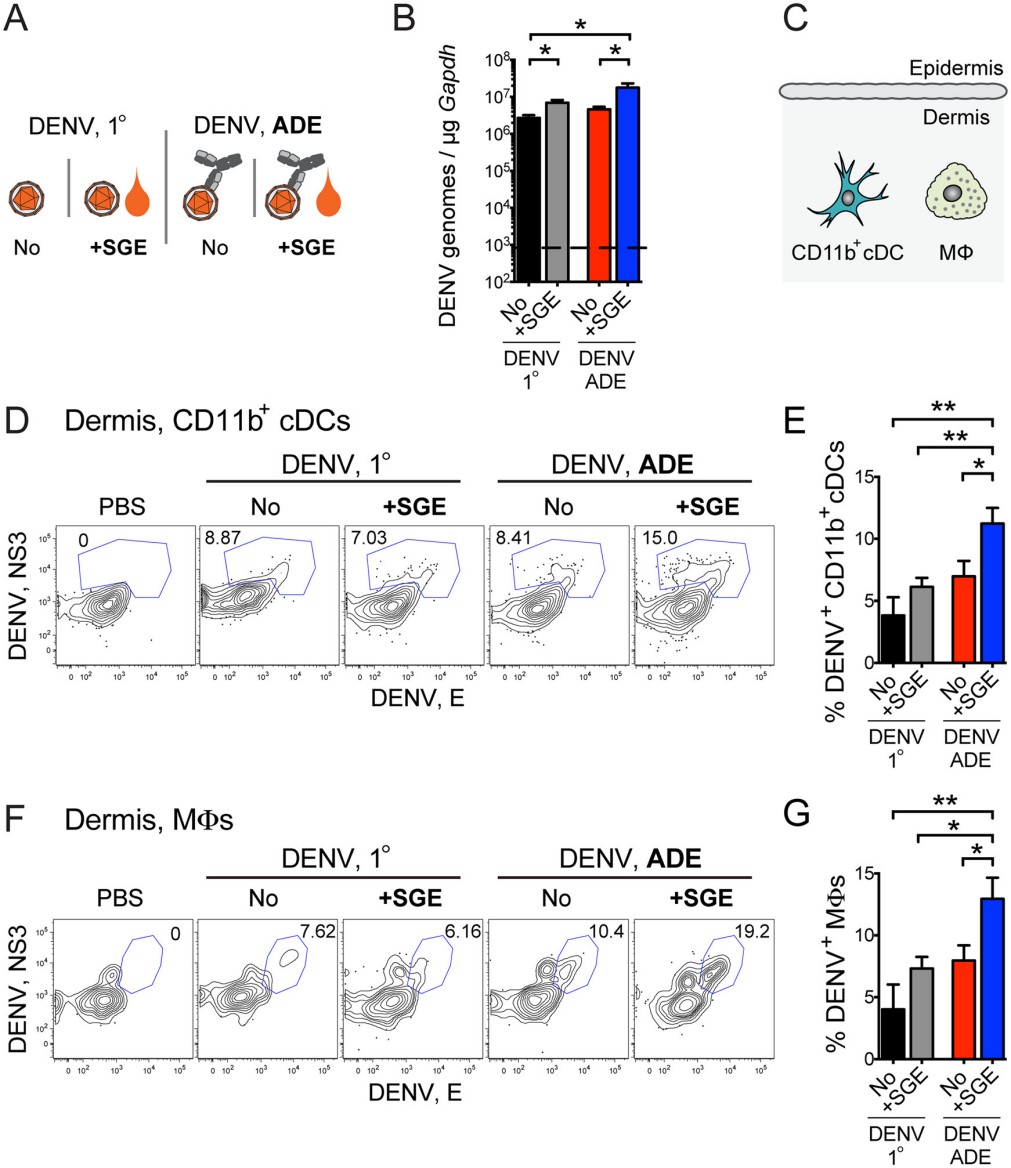
SGE increases DENV infection in the skin during ADE. (A) *Ifnar* ^–/–^mice were inoculated i.d. with 10^5^ PFU DENV under 1° or ADE conditions in the presence or absence of SGE. (B) Bar graph showing mean ±SEM DENV genome equivalents in the skin after 14 h normalized to 1 μg *Gapdh* mRNA, as determined by qRT-PCR (limit of detection depicted as horizontal dashed line). Data were pooled from two experiments, n = 6 per group. Statistically significant differences are marked as * for p<0.05. (C) Schematic of the main dermis-resident immune cells. (D) Contour plots showing intracellular staining for DENV non-structural protein NS3 and structural protein E of dermal CD11b^+^ cDCs analyzed via flow cytometry 14 h after inoculation. For gating of cell populations, see S2 Fig. (E) Bar graph summarizing percent DENV infection of CD11b^+^ cDCs. (F) Contour plots showing intracellular staining for DENV proteins of dermal MΦs. (G) Bar graph summarizing percent DENV infection of dermal MΦs. Data are representative (D, F) or pooled (E, G) from three independent experiments, n = 6–9 per group. Statistically significant differences are marked as * for *p*<0.05 and ** for p<0.01.

We hypothesized that SGE synergizes with enhancing antibodies to impact DENV replication in the skin. In fact, SGE alone and to a greater degree the combination of SGE and enhancing antibodies significantly increased DENV titers in the skin at 14 h post-inoculation, as measured by qRT-PCR (Fig 2A and 2B). We thus dissected DENV infection of cell subsets in the skin via intracellular staining for DENV non-structural protein NS3 and structural protein E in flow cytometric analysis. We gated dermis-resident CD11b^+^ cDCs (CD45^+^ MHCII^high (hi)^ Langerin^-^CD11b^+^ Ly6C^-^) and MΦs (CD45^+^ MHCII^low/–^Langerin^-^CD11b^hi^ Ly6G^-^ Ly6C^low/–^SSC^int-hi^) as previously established ([28] and S2 Fig). Dermis-resident CD11b^+^ cDCs and MΦs were the initial targets for DENV infection 14 h after i.d. inoculation of 10^5^ PFU (Fig 2C–2G), similar to inoculation of 10^6^ PFU, as reported previously [28]. While DENV infection was low in the absence of SGE or enhancing antibodies, the combined action of SGE and enhancing antibodies significantly increased DENV infection of dermal CD11b^+^ cDCs (Fig 2D and 2E) and MΦs (Fig 2F and 2G). While myeloid cells in the dermis expressed various levels of Fc*γ* receptors (CD16/32), only MΦs and, to a minimal degree, CD11b^+^ cDCs expressed mannose receptor (CD206, S2 Fig). These dermal cell surface molecules may serve to mediate DENV attachment in the presence (Fc*γ* receptors) [8,42] or absence (mannose receptor for MΦs) of enhancing antibodies [43]. No cells in the epidermis, such as CD45^+^ MHCII^+^ Langerin^+^ Langerhans cells, contained intracellular DENV proteins after 14 h. The combined presence of mosquito-derived factors and enhancing antibodies thus augmented DENV infection of CD11b^+^ cDCs and MΦs as initial targets for DENV replication in the dermis.

### SGE amplifies neutrophil and monocyte recruitment to the dermis

**Fig 3:**
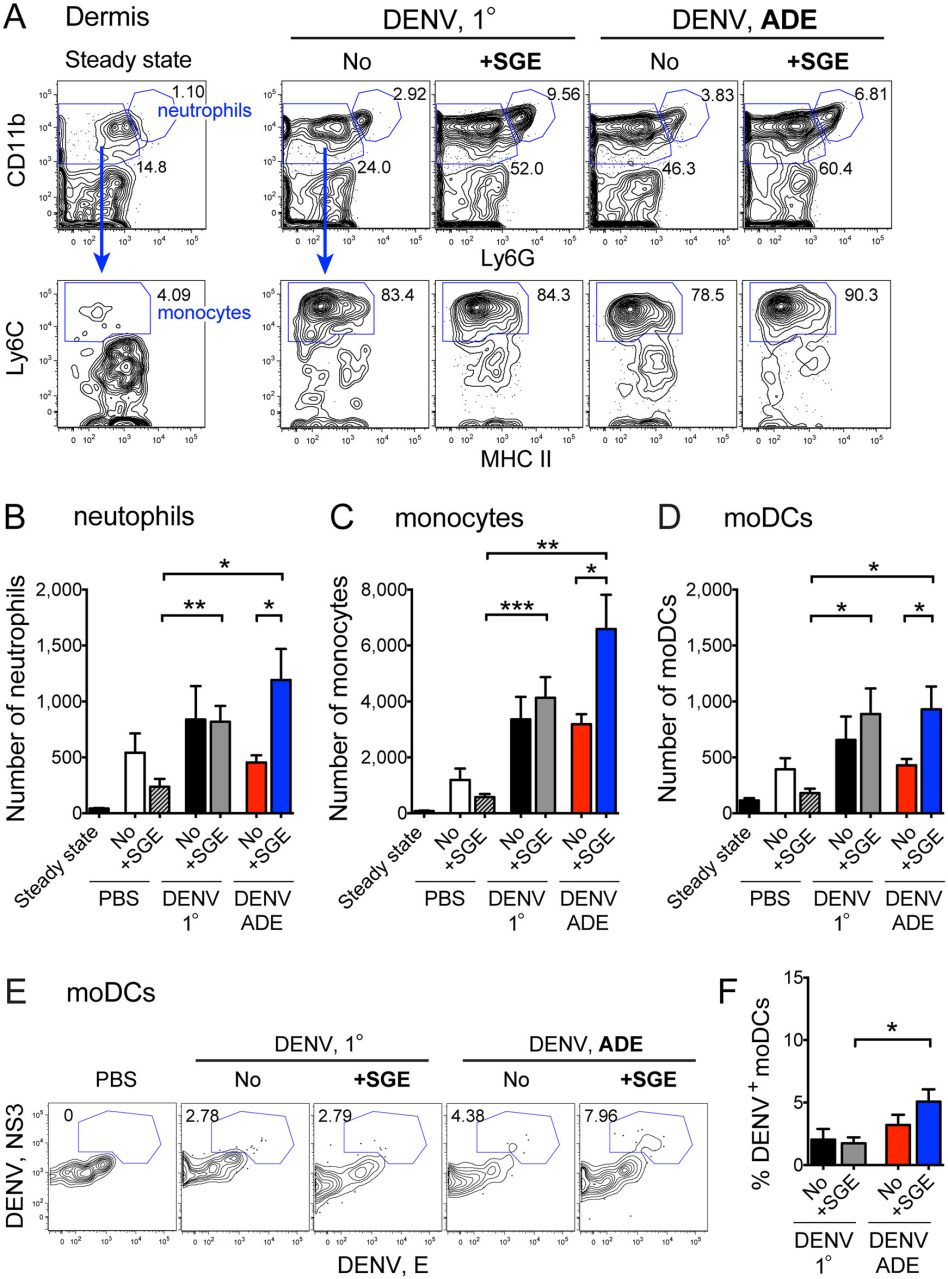
SGE together with enhancing antibodies augments recruitment of neutrophils and monocytes to the dermis. (A) Contour plots showing consecutive gating of neutrophils and monocytes among CD45^+^ MHCII^low/–^cells in the dermis, as determined via flow cytometry of steady-state mice or 14 h after i.d. inoculation of 10^5^ PFU DENV in the presence or absence of SGE under 1° or ADE conditions. Numbers on plots are percent of gated cells of the parent population. (B-D) Bar graphs showing mean ±SEM number of neutrophils (B), monocytes (C) or moDCs (D) in the dermis, gated as in (A) and S2 Fig. (E) Contour plots showing intracellular staining for DENV proteins of moDCs in the dermis. (F) Bar graph showing mean ±SEM percent DENV infection of dermal moDCs. Data are representative (A, E) or pooled (B-D, F) from three experiments, n = 6–9 per group. Statistically significant differences are marked as * for p<0.05, ** for p<0.01, and *** for p<0.001.

We next examined the recruitment of immune cells to the dermis. We gated *de novo*-recruited cell populations in the dermis as previously established via adoptive transfer of labeled monocytes in the absence of SGE [28]. Ly6G expression identified neutrophils, and Ly6C expression separated recruited Ly6C^hi^ monocytes from resident Ly6C^low/–^MΦs among MHCII^low/–^CD11b^+^ cells (Figs 3A and S2). Whereas inoculation of 10^5^ PFU DENV under 1° conditions led to a modest recruitment of inflammatory neutrophils (Fig 3B) and monocytes to the dermis (Fig 3C), SGE significantly amplified the recruitment of neutrophils and monocytes in the presence of enhancing antibodies. Ly6C expression further separated Ly6C^+^ moDCs from resident Ly6C^-^ CD11b^+^ cDCs among MHCII^+^ Langerin^-^CD11b^+^ cells (S2 Fig). During ADE, SGE augmented the number (Fig 3D) and DENV infection (Fig 3E and 3F) of *de novo*-recruited moDCs in the dermis. In contrast, few monocytes (<0.5%) and no other cell types (e.g., DCs or MΦs) were infected with DENV in the dermis 14 h after inoculation. SGE also increased the percentage of monocytes and moDCs in the dermis (S3 Fig). Together, these data show that the presence of SGE and enhancing antibodies augmented innate responses to DENV infection in the skin via the recruitment of neutrophils, monocytes, and moDCs and increased the infection of *de novo*-recruited moDCs.

### SGE increases dendritic cell migration to skin-draining lymph nodes

**Fig 4:**
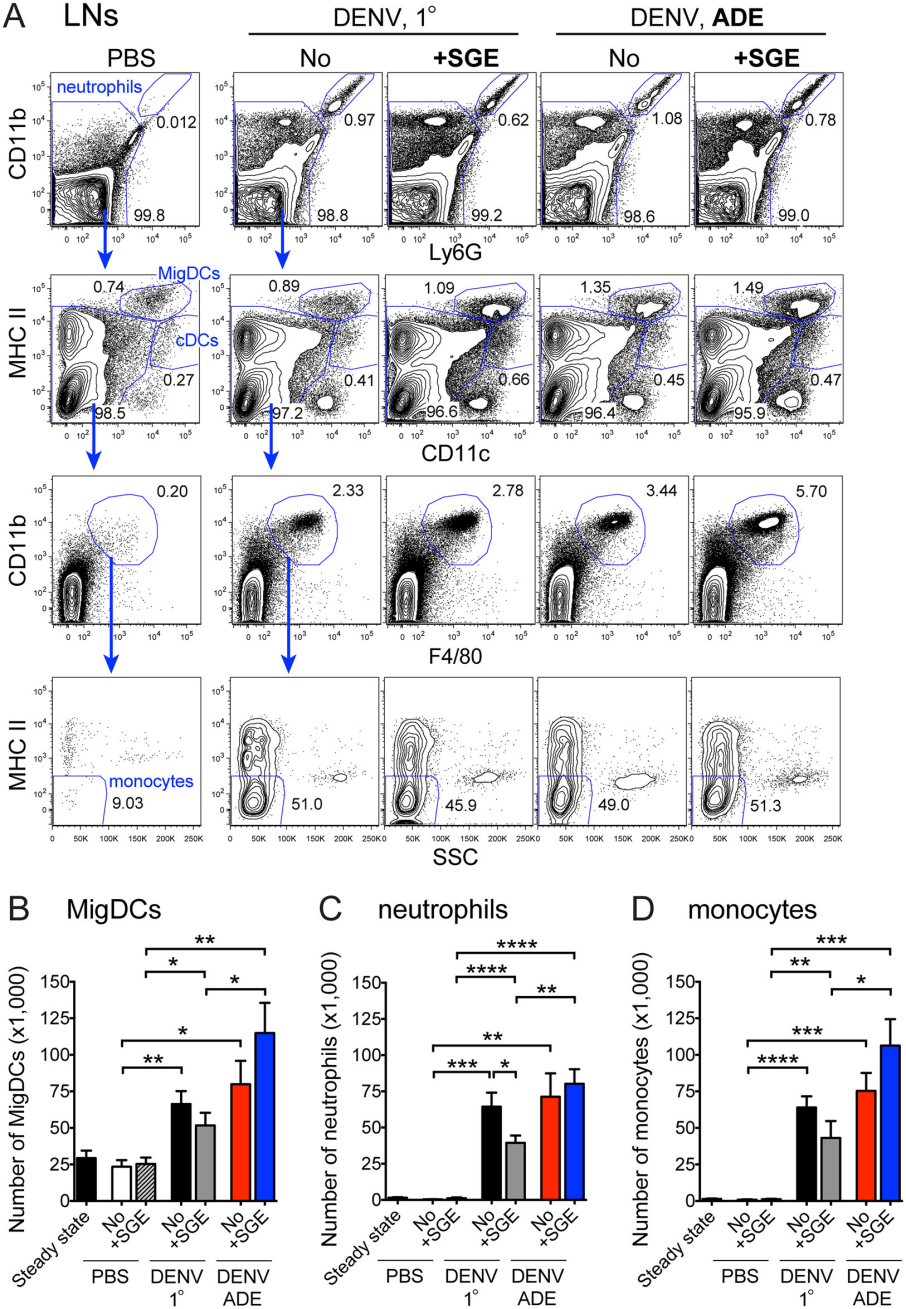
SGE accelerates migration of dendritic cells and recruitment of neutrophils and monocytes to lymph nodes during ADE. Mice were inoculated i.d. with PBS or 10^5^ PFU DENV under 1° or ADE conditions in the presence or absence of SGE. (A) Contour plots showing cells in LNs 14 h after inoculation. Consecutive gating identified neutrophils as Ly6G^+^ CD11b^hi^, MigDCs as Ly6G^-^ CD11c^int^ MHCII^hi^, cDCs as Ly6G^-^ CD11c^hi^ MHCII^int^, and monocytes as Ly6G^-^ CD11c^–/low^ MHCII^-^CD11b^+^ F4/80^+^. For expression of CCR7 and Fc*γ* receptor, see S4 Fig. Bar graphs summarizing the number of MigDCs (B), neutrophils (C) or monocytes (D) in LNs. Data are representative (A) or pooled (B-D) from three experiments, n = 6–9 per group. Statistically significant differences between groups are marked as * for p<0.05, ** for p<0.01, *** for p<0.001, and **** for p<0.0001.

We hypothesized that SGE may further modulate immune responses beyond the skin. While activated cDCs efficiently migrate from the skin to draining LNs (MigDCs) via expression of the chemokine receptor CCR7 [44,45] and prime naïve T and B cells, MΦs and recruited moDCs mostly remain within the inflamed skin to phagocytose pathogens and locally stimulate effector or memory T cells [46,47]. After 14 h, skin-draining LNs contained significantly more and a higher frequency of MigDCs with a CD11c^int^ MHCII^hi^ CCR7^+^ migratory phenotype (Figs 4A and S4) when DENV was inoculated with SGE in the presence of enhancing antibodies (Fig 4B). The combined presence of SGE and enhancing antibodies further boosted the recruitment of CD11b^hi^ Ly6G^+^ neutrophils (Fig 4C) and MHCII^-^CD11b^+^ F480^+^ SSC^low^ monocytes to LNs (Fig 4D), likely from the blood. For neutrophils, this effect was due to decreased recruitment during 1° infections when SGE was present. In contrast, SGE did not significantly change the number of CD11c^hi^ MHCII^int^ cDCs or cells with a CD11c^low^ MHCII^int^ CD11b^+^ F480^+^ SSC^low^ phenotype that likely consist of moDCs in LNs after 14 h (S4 Fig). We further examined the activation of naïve T cells during antibody-enhanced DENV infection. Even though 82% of CD8^+^ and 58% CD4^+^ T cells in the spleen and 34% of CD8^+^ and 21% CD4^+^ T cells in LNs draining the site of i.d. inoculation acquired an activated CD44^+^ CD62L^-^ phenotype within 5.5 days, no significant differences were detectable in the presence or absence of SGE (S5 Fig). Along the same lines, 22% of CD8^+^ T cells and 13% of CD4^+^ T cells produced IFN-*γ* in the spleen regardless of whether SGE was present or not (S5 Fig). However, a non-significant trend towards decreased proliferation of T cells in the presence of SGE was observed (S5 Fig). Consequently, the presence of SGE during antibody-enhanced DENV infection increases the migration of DCs and monocytes to skin-draining LNs. Nevertheless, whether augmented innate responses subsequently affect adaptive immune responses and thereby influence dengue pathogenesis remains to be determined.

### SGE disrupts endothelial barrier function

**Fig 5:**
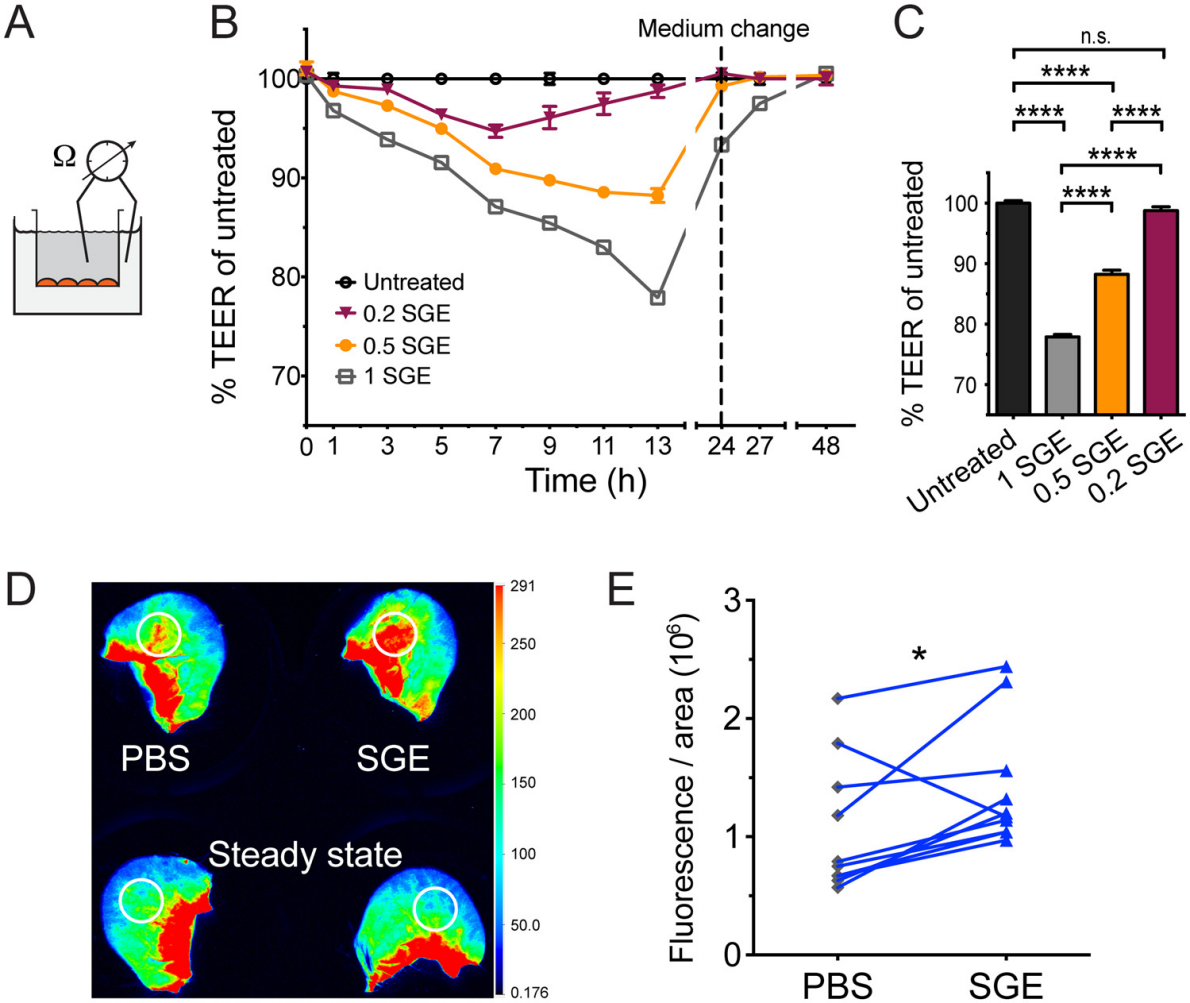
SGE disrupts endothelial barriers *in vitro* and *in vivo* in the skin. (A-C) The trans-endothelial electrical resistance (TEER) of human microvascular endothelial cell (HMEC-1) monolayers grown in Transwell chambers were measured as depicted in (A) after adding the equivalent of 0.2, 0.5 or 1 salivary glands to the basolateral compartment. (B) Graph showing percent TEER relative to untreated control cells over time. (C) Bar graph depicting percent TEER after 13 h of stimulation. Data were pooled from two experiments, n = 8 per group. Statistically significant differences are marked as **** for *p*<0.0001 or not significant (n.s. for p>0.05). (D-E) Wild-type mice were inoculated intravenously with fluorescently-labeled dextran, and SGE or PBS was inoculated i.d. into either ear of each animal immediately thereafter. Steady-state ears of dextran-inoculated control mice were left untouched. (D) Representative scan of ears analyzed after 30 min showing dextran fluorescence that leaked with plasma into the dermis at sites of i.d. inoculation or corresponding areas in steady-state ears (white circles). (E) Mean fluorescence of dextran per an area of 3300 pixels of ear skin ±SEM quantified at the site of i.d. inoculation with PBS or SGE for each animal (connecting lines). Data were pooled from three experiments, n = 9 per group. Statistically significant differences between PBS and SGE are marked as * for *p*<0.05.

Endothelial cells lining blood vessels separate the skin from systemic blood circulation. We hypothesized that factors contained in SGE may disrupt endothelial barrier function to enable blood feeding, which might also facilitate cell migration. To determine the direct effect of SGE on the vascular endothelium, we measured trans-endothelial electrical resistance (TEER) of human dermal microvascular endothelial cell (HMEC-1) monolayers growing on Transwell inserts (Fig 5A). Monolayers displayed high TEER values and thus low permeability in steady state. Addition of SGE to either the basolateral side of the monolayer that corresponds to the side facing the dermis (Figs 5B, 5C and S6) or the apical side facing the blood (S6 Fig) significantly decreased TEER values, thus increasing endothelial permeability, in a dose-dependent manner. TNFα served as a positive control for endothelial permeability and decreased TEER values with similar dynamics as SGE (S6 Fig). We next tested whether SGE also affected endothelial barrier function at the site of inoculation in the mouse ear. Our results demonstrated that i.d. inoculation of SGE disrupted the barrier function of the microvasculature in the mouse ear and increased the leak of plasma into the dermis, visualized via fluorescently-labelled dextran that was inoculated intravenously (Fig 5D). The vasculature in the PBS-inoculated control ear of the same animal showed significantly lower leak of plasma, and untouched ears of steady-state animals showed only minor endothelial permeability (Fig 5D and 5E). The signal at the base of the ear was due to autofluorescence of cartilege and was excluded from quantification at the site of inoculation (white circles, Fig 5D). Consequently, SGE disrupts endothelial barrier function at the site of virus transmission in the dermis and may thus be one factor that contributes to systemic dengue pathogenesis.

### The effect of SGE spreads rapidly beyond the skin and modulates dengue pathogenesis

**Fig 6:**
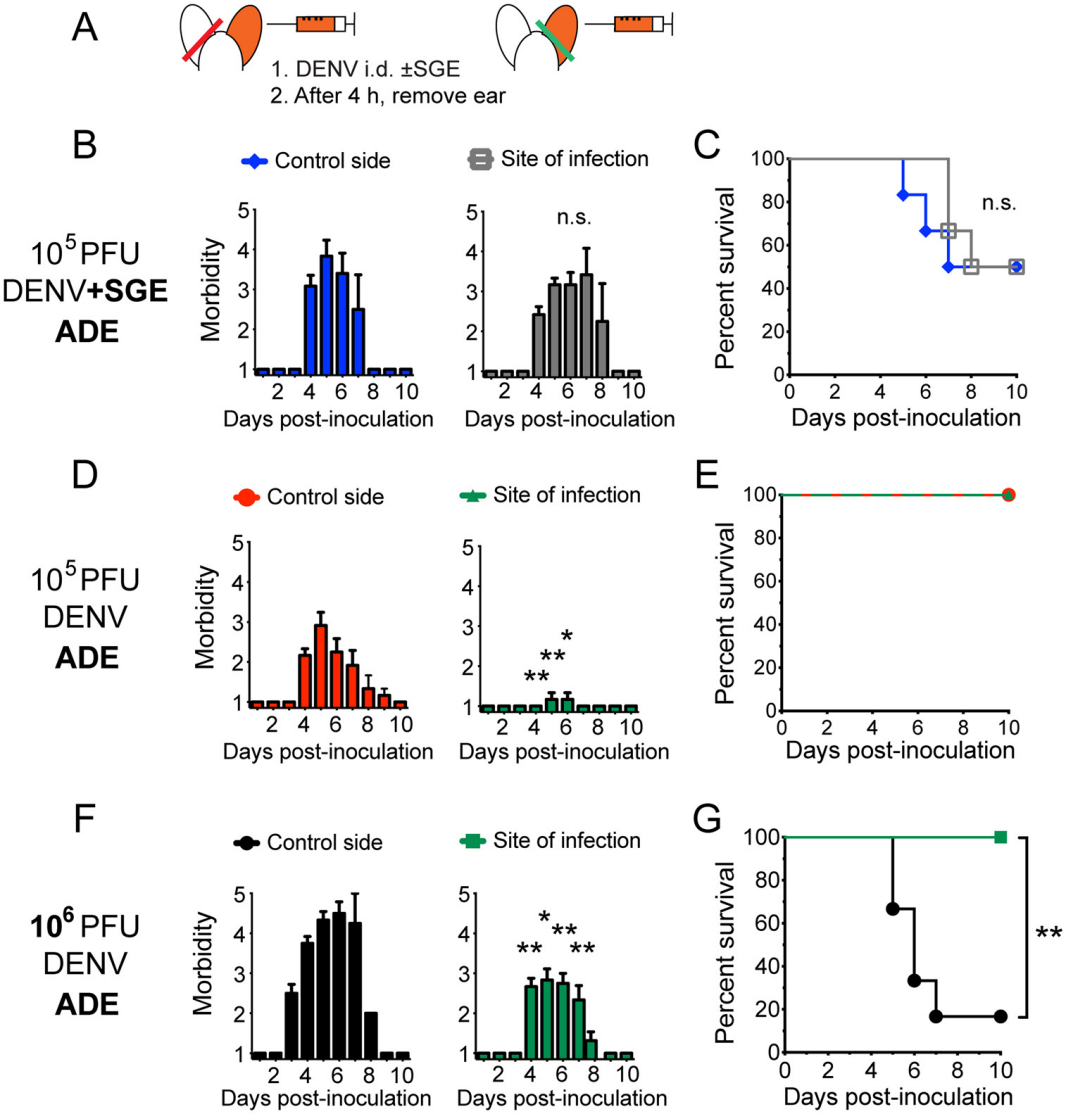
Removing the site of DENV inoculation in the skin no longer rescues mice from dengue disease in the presence of SGE. (A) *Ifnar* ^–/–^mice were inoculated i.d. with DENV under antibody-enhanced conditions, and 4 h later, the non-inoculated control side or the site of inoculation in the ear skin was surgically removed. (B) Bar graphs showing mean morbidity ±SEM and (C) Kaplan-Meier curve showing survival of mice infected with 10^5^ PFU DENV mixed with SGE. (D) Bar graphs showing morbidity and (E) Kaplan-Meier curve showing survival of mice infected with 10^5^ PFU DENV alone. (F) Bar graphs showing morbidity and (G) Kaplan-Meier curve showing survival of mice infected with 10^6^ PFU DENV alone. Data were pooled from two experiments, n = 6 per group. Statistically significant differences between removing the site of DENV inoculation and removing the non-inoculated control side are marked as * for p<0.05 or ** for p<0.01.

Finally, to test whether the effects of SGE in the skin impacted systemic dengue pathogenesis, we surgically removed the site of DENV transmission in the presence or absence of SGE after 4 h (Fig 6A). In the presence of SGE, removing the site of DENV transmission did not have a protective effect on the morbidity and survival of mice after i.d. inoculation with an antibody-enhanced DENV inoculum of 10^5^ PFU (Fig 6B and 6C). Fifty percent of mice succumbed to infection regardless of whether the control side or the site of inoculation was removed. In contrast, if no SGE was present, removing the site of DENV transmission completely protected mice from morbidity after inoculation with an antibody-enhanced dose of 10^5^ PFU (Fig 6D and 6E). Even more striking was the significant protective effect on morbidity and survival of the mice observed after inoculation with an antibody-enhanced high DENV dose of 10^6^ PFU (Fig 6F and 6G). These data show that the effect of mosquito-derived factors on dengue pathogenesis rapidly spreads beyond the skin. The effect of SGE on endothelial barrier function and the immune response thus influence dengue pathogenesis.

## Discussion

We establish here that *Ae*. *aegpyti* SGE exacerbates systemic dengue pathogenesis during ADE. We determined that SGE in the presence of enhancing antibodies increases DENV infection of cDCs and MΦs in the dermis, recruitment of monocytes to the dermis and draining LNs, and DC migration from the skin to LNs. We also showed that SGE acts directly on endothelial cells *in vitro*, disrupts endothelial barrier function, and induces plasma leak in the mouse ear. Further, surgical removal of the site of DENV transmission after 4 h rescued mice from enhanced disease in the absence but not in the presence of SGE, indicating that the effect of mosquito-derived factors on dengue pathogenesis spreads rapidly beyond the skin.

Previous studies have demonstrated that mosquitoes inoculate saliva that contains pathogens into the dermis while probing for blood vessels. *Anopheles* mosquitoes inoculated saliva with *Plasmodium* parasites into the dermis of mice [48]. Similarly, in studies of mosquitoes feeding on mouse tails, >99% of inoculated WNV was recovered from the skin section that infected *Culex* mosquitoes probed upon [41]. Although *Culex* mosquitoes inoculated WNV directly into the blood circulation when obtaining a successful blood meal [41], the amount of WNV that was deposited into the bloodstream remained low at ~100 PFU, which was only 0.1% of the total i.d.-transmitted median dose of 10^5^ PFU of WNV [41]. We show that removing the site of i.d. DENV infection after 4 h rescued mice from disease in the absence of SGE but, in the natural setting, DENV is always transmitted in the saliva of infected mosquitoes. Studies have shown that survival of mice was prolonged when the distal third of the tail was removed 5 min to 6 h after mosquitoes inoculated with St. Louis encephalitis [49] or Rift Valley fever viruses [50]. Similarly, mice were protected from blood parasitemia when the site of inoculation was removed up to 5 min but no later than 15 min after *Anopheles*-transmitted *Plasmodium* infection [51]. Our data that removing the site of DENV transmission rescues mice in the absence but not in the presence of SGE establishes that DENV infection in the skin early after inoculation is critical for subsequent dengue pathogenesis and that SGE modulates the dynamics of this process.

The endothelial barrier of capillaries separates the skin from systemic blood circulation. Mosquito saliva [1,18] or innate immune recognition of DENV [52,53] can increase endothelial cell permeability via activation of mast cells and release of inflammatory mediators, such as histamine, TNFα, or proteases. Our *in vivo* results of SGE inducing vascular leak in the ear skin may thus be a combined effect of SGE on mast cells and endothelial cells. In contrast, our *in vitro* results of SGE decreasing the TEER of endothelial cell monolayers demonstrate that SGE can act directly on endothelial cells to increase permeability. In the absence of SGE, Luplertlop *et al*. showed that DENV-infected DCs induce vascular permeability in the skin via production of metalloproteinases that degrade endothelial cell adhesion molecules [54]. Serine proteases in *Ae*. *aegypti* SGE were shown to degrade the extracellular matrix of embryonic fibroblasts [40]. We thus speculate that SGE disturbs the integrity of blood vessels through degradation of extracellular matrix proteins or disturbance of tight/adherens junctions between endothelial cells. Factors in SGE that disrupt endothelial barrier function may have evolved to help the mosquitoes penetrate blood vessels or feed from a pool of blood in the dermis after injury to a blood vessel.

Previous studies examined the effect of mosquito-derived factors on DENV infection only in the absence of enhancing antibodies [24,25,40]. In our hands, SGE did not augment dengue pathogenesis after inoculation of 10^5^ PFU under 1° conditions and even reduced disease severity after inoculation at a 10-fold higher dose of 10^6^ PFU. In contrast, SGE augmented dengue pathogenesis during antibody-enhanced infection with 10^5^ PFU. Numerous reports show that ADE enhances DENV infection of human monocytes [8,55] and MΦs [9] *in vitro*. In mice, intestinal monocytes/MΦs and liver sinusoidal endothelial cells are particularly susceptible to antibody-enhanced DENV infection [15,30]. We speculate that, via disruption of endothelial barrier function in the skin, SGE may augment access of DENV to enhancing antibodies in the serum, increase the spread of infectious virus-antibody complexes, enhance infection within systemic tissues, and thus exacerbate dengue pathogenesis during ADE. Our results thus suggest that animal models of dengue and pre-clinical validation of dengue vaccine candidates should be evaluated in the combined presence of mosquito saliva and enhancing antibodies.

As one possibility, SGE may augment systemic dengue pathogenesis by disrupting endothelial barrier function and thus facilitating DENV dissemination. In humans, higher DENV titers at peak viremia correlate with more severe disease [5]. Accelerated early DENV dissemination may subsequently increase virus titers and thus the risk for severe disease. In mice, feeding of uninfected *Culex* mosquitoes at the site of needle inoculation or concurrent inoculation with SGE augmented WNV serum viremia after 24–48 h [22]. Likewise, feeding of uninfected *Ae*. *aegypti* at the site of needle inoculation with WNV subsequently increased mortality compared to needle inoculation with virus alone [23]. Similarly, mosquito transmission of DENV or needle-inoculation with concurrent feeding of uninfected mosquitoes prolonged viremia and increased erythema (redness of the skin) in “humanized” mice [25] and increased DENV titers around peak viremia in mice lacking IRF3/7 [24,56]. When needle-inoculated into the foot pad, mosquito SGE further increased DENV titers in LNs of *Ifnar* ^–/–^mice after 24 h [40]. Together, these data show that mosquito-derived factors increase DENV titers 24 h to several days post-inoculation, which could be due to increased virus dissemination and/or systemic replication. Our ear removal data show that factors that augment dengue pathogenesis spread beyond the skin within 4 h in the presence of SGE. Nevertheless, the precise impact of mosquito-derived factors on early DENV dissemination prior to the first round of DENV replication, which takes place within ~12 h [57], remains unknown.

Our data also show that SGE boosts the recruitment of innate immune cells during acute DENV infection. Previous studies revealed that monocytes and neutrophils enter the DENV-infected dermis in the absence of SGE [28]. Several studies have confirmed the recruitment and phenotype of inflammatory monocytes (MHCII^-^Langerin^-^CD11b^+^ Ly6G^-^ Ly6C^hi^) and moDCs (MHCII^hi^ Langerin^-^CD11b^+^ Ly6C^+^) to the dermis during allergic reactions [58], or infection with *Leishmania major* [59] or DENV [28] via adoptive transfer of genetically- or fluorescently-labeled monocytes. The phenotype remained consistent for 7 days, before monocyte-derived cells down-regulated Ly6C expression [58,59]. During DENV infection, monocyte recruitment and differentiation to moDCs increased the number of targets for virus replication in the dermis [28]. Sterile injury [60] or transmission of *Leishmania* via sandfly feeding triggered the recruitment of neutrophils to the skin, where they then served as a “Trojan horse” reservoir for parasite infection [61], and abrogated vaccine-induced protection by suppressing parasite-specific CD4^+^ T cell responses [62]. Few studies have addressed the role of neutrophils in dengue pathogenesis. In one study, the serum of dengue patients contained higher levels of IL-8 and neutrophilic elastase than healthy controls, suggesting activation and degranulation of neutrophils [63]. Previous studies have documented the exit of epidermal Langerhans cells [64,65] and dermal DCs [27] from DENV-infected skin and their migration to LNs in the absence of SGE. We establish here that the combined presence of enhancing antibodies and SGE boost the recruitment of monocytes and neutrophils to the DENV-infected dermis and draining LNs, as well as DC migration from the skin to LNs. SGE disruption of endothelial barrier function in the dermis may facilitate both transmigration of monocytes and neutrophils through blood vessels to enter the dermis and migration of skin DCs to LNs via increased drainage of plasma that leaks into the skin. Because removal of the site of co-inoculation of DENV with SGE after 4 h did not rescue mice from systemic antibody-enhanced pathogenesis, rapid spread of inflammatory mediators may contribute to increased disease severity. As we did not observe significant differences in T cell activation in the presence versus absence of SGE 5.5 days after DENV inoculation, further studies are needed to determine whether SGE affects systemic immune responses. Along the same lines, Cox *et al*. determined that increased viremia and prolonged fever occurred even when mosquito saliva and DENV were inoculated at distant sites [25], supporting the view that mosquito-derived factors can impact dengue pathogenesis systemically.

We confirm here the recent discovery that dermal cDCs and MΦs are the initial targets for DENV replication in the skin of mice and humans in the absence of SGE [27,28], but at a 10-fold lower virus dose *in vivo*. Only one *in vitro* study has examined the impact of mosquito saliva on human immature moDCs, and it found that *Ae*. *aegypti* saliva inhibits DENV infection under 1° conditions [26]. In our study, SGE led to a small but significant increase in DENV titer in the skin under 1° conditions, as measured via qRT-PCR 14 h after inoculation. Under 1° conditions, however, flow cytometric analysis did not detect significant differences in the infection of dermal cDCs, moDCs, and MΦs, which were the only DENV-infected cells in the skin at this time-point. We establish here that the combined presence of SGE and enhancing antibodies significantly enhanced DENV infection of cDCs and MΦs in the dermis. While monocytes, MΦs, and mature moDCs are known targets for enhanced DENV infection *in vitro* [8,9,42], this is the first report that ADE boosts DENV infection of cDCs in the presence of SGE. In contrast, ADE alone did not significantly enhance infection of cDCs and MΦs in the dermis in the absence of SGE (Fig 2 and [28]). Whereas enhancing antibodies may have limited access to the skin in the absence of SGE, the increased leak of plasma into the skin that we found in the presence of SGE may allow more enhancing antibodies to enter the skin and mediate antibody-enhanced infection of cDCs and MΦs in the dermis. Although DCs are key for priming adaptive T and B cell responses, DENV infection can impair DC function [10]. In addition, *Ae*. *aegypti* saliva suppresses host responses, such as cytokine production and T cell proliferation at the feeding site [66,67], and skews systemic CD4^+^ T cell responses from Th1- towards Th2-type by decreasing IFN-*γ* and increasing IL-4 expression [56,68,69]. Even though our ear removal experiments show that dengue pathogenesis of the ongoing infection occurs beyond the skin when SGE and enhancing antibodies are present, the increased DENV infection of skin DCs may affect priming of memory responses that protect or enhance pathogenesis during subsequent DENV infections.

In endemic regions, exposure to non-infected arthropod vectors regularly occurs and could modulate the effect of vector saliva. For malaria, exposure of mice to the bite of non-infected *Anopheles* mosquitoes protected them from subsequent mosquito-transmitted *Plasmodium* infection [70]. Similarly, vaccination with sand fly SGE or exposure to the bites of non-infected sand flies protected mice or non-human primates against subsequent *Leishmania* challenge plus SGE [71–74]. However, immunization with two distinct sand fly salivary proteins, PpSP15 or PpSP44, either protected or increased susceptibility to *Leishmania* infection, respectively [75]. Similarly, pre-exposure of mice to bites of non-infected *Ae*. *aegypti* mosquitoes exacerbated WNV lethality [76], but immunization with the recombinant protein D7 from *Culex* saliva enhanced pathogenesis during subsequent mosquito-transmitted WNV infection [77]. One study reported an association of higher serum reactivity to *Ae*. *aegypti* salivary proteins in Thai children with severe secondary dengue disease compared to not-infected individuals, but it remains unclear whether this correlation was due to prior exposure or reactivity to DENV or non-infected mosquitoes [78]. In our hands, antibody-enhanced DENV infection in the presence of SGE induced lethality in a comparable proportion of mice that were pre-exposed three times to SGE versus SGE-naïve animals. Further studies are needed to determine the effect of anti-*Ae*. *aegypti* immunity on dengue pathogenesis, and the potential use of mosquito-derived components in vaccine formulations against dengue requires thorough evaluation.

Our study introduces *in vitro* and mouse models to study the impact of mosquito-derived factors on endothelial cells and dengue pathogenesis, respectively. DENV suppresses the IFN response, replicates, and causes disease in humans but not wild-type mice [28,35,36]. *Ifnar* ^–/–^mice are readily available and more immunocompetent than other DENV infection models due to intact IFN-*γ* receptor signaling. After i.d. inoculation, DENV infects the same cells (i.e., dermal cDCs and MΦs) in *Ifnar* ^–/–^mice [28] and human skin explants [27], cross-validating the reliability of these models. Studies using transmission via DENV-infected live mosquitoes are most physiologically relevant but naturally cannot control for the presence of mosquito-derived factors or the virus dose delivered, as mosquitoes transmit varying doses of virus (e.g., WNV [41]). A study design (“spot feeding”) where uninfected female *Ae*. *aegypti* mosquitoes deposit saliva into mouse skin followed by i.d. needle inoculation of virus mimics the secretion of saliva while mosquitoes probe for blood vessels, can control for the presence or absence of mosquito-derived components, delivers a defined dose of virus, and has successfully been used to study DENV infection *in vivo* [24,25]. Live mosquitoes, however, inoculate varying amounts of saliva [41] depending on how fast they locate and penetrate a blood vessel; thus, salivary dose cannot be controlled or monitored in this model. The simplified model that we use here, i.e., needle inoculation of SGE from non-infected mosquitoes with a defined dose of pathogen, has proven useful in assessing the role of vector-derived factors for infection with DENV [40], WNV [23], Sindbis virus [69], Rift Valley fever virus [39], and *Leishmania* [38]. Similarly, WNV transmission via infected *Culex* mosquitoes, deposition of saliva via uninfected mosquitoes at the site of needle inoculation, or concurrent i.d. needle inoculation of WNV with SGE all induced earlier and higher viremia compared to needle inoculation of WNV alone [22][79]. While not precisely replicating DENV transmission via live mosquitoes, the SGE model is more broadly applicable to future immunological analyses and pre-clinical testing of dengue vaccines, as the variables can be controlled, unlike mosquito-transmitted DENV. Mosquito saliva can be collected artificially in a sucrose solution in a capillary tube into which the mosquito proboscis is inserted, or through a membrane. However, such saliva preparations differ qualitatively and quantitatively from mosquito saliva that is inoculated into the host during a natural blood meal [79,80]. Furthermore, the mosquito does not replicate normal probing and secretion of saliva during artificial feeding. Needle inoculation i.d. of DENV with or without SGE is thus the model that is easiest to standardize between assays and between research groups. The dose of 1 SGE that we have used *in vivo* contained 0.37–0.60 μg total protein and lies within the range for which Moser *et al*. determined a dose-dependent increase in WNV serum viremia in the presence of SGE from *Culex* mosquitoes [79]. In addition, Wasserman *et al*. determined that a single *Ae*. *aegypti* mosquito inoculates approximately 0.35 salivary gland pairs (equivalent to 0.7 SGE) [81]. We have thus inoculated mice with SGE that is likely equivalent to the bite of 1–2 mosquitoes. We further introduce an *in vitro* system measuring TEER as a practical tool to assess the direct effect of vector-derived factors on endothelial barrier function that is independent of specific pathogens and can thus be extrapolated to other viruses carried by *Ae*. *aegypti* mosquitoes. Using the TEER system, we show that 0.2 to 2 SGE increased vascular permeability in a dose-dependent manner, indicating that 1 SGE is a non-saturated dose but lies within the range of a dose-response curve.

**Fig 7:**
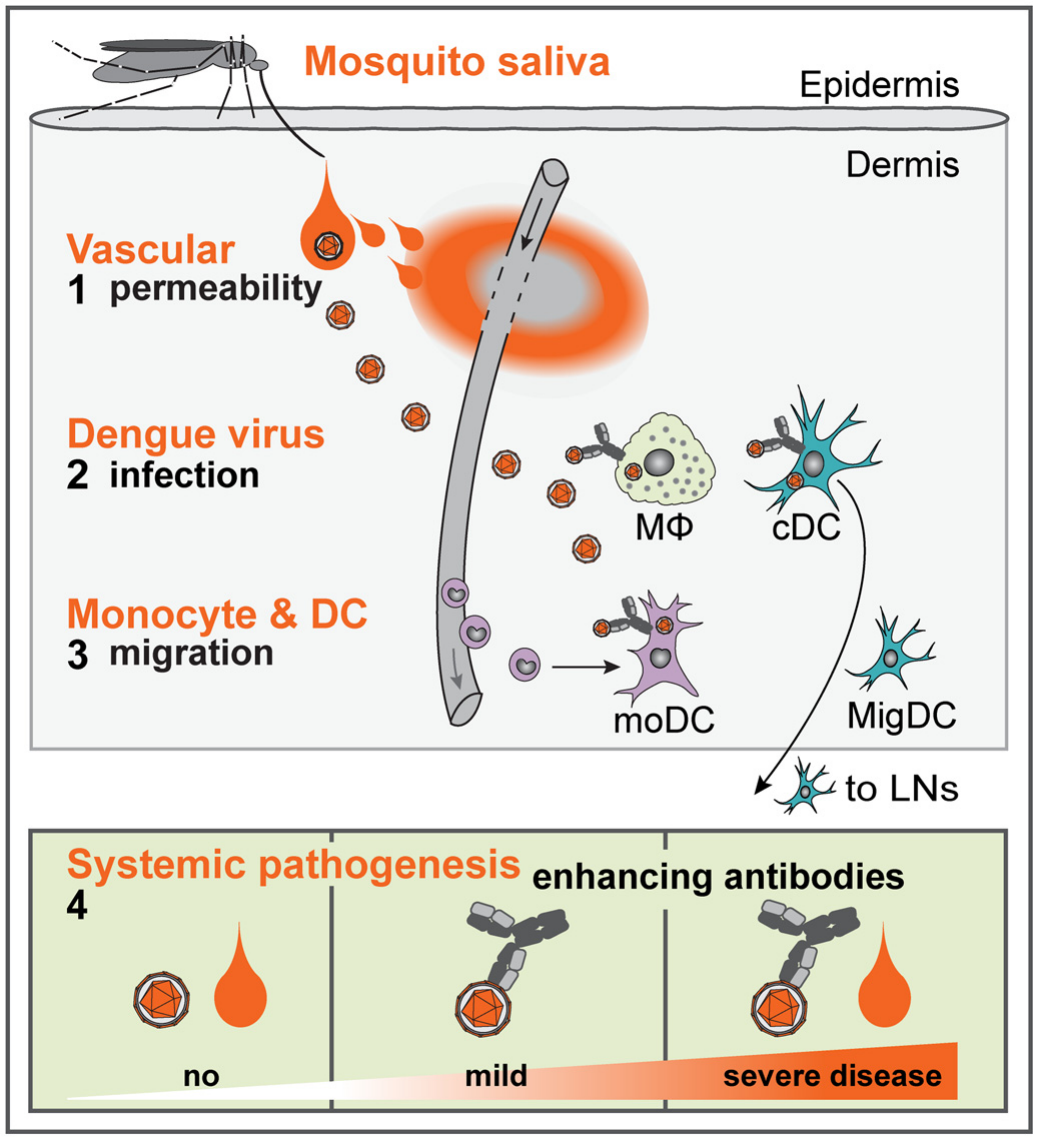
The effects of mosquito SGE in the skin and on systemic dengue pathogenesis. In summary, our data show that (1) SGE induces endothelial permeability and vascular leak in the mouse ear. During ADE, SGE further increases (2) DENV infection of MΦs and cDCs that reside in the dermis. In addition, (3) SGE boosts monocyte recruitment to the dermis and draining LNs, differentiation to moDCs, and migration of DCs (MigDCs) from the skin to LNs. During systemic pathogenesis (4), only the combined presence of SGE and enhancing antibodies leads to severe disease.

In summary (Fig 7), our study establishes that mosquito-derived factors exacerbate dengue pathogenesis in individuals that carry enhancing antibodies and thus already have an increased risk for severe disease. Consequently, safety and efficacy of vaccine and therapeutic candidates against dengue should be tested pre-clinically in models that consider both the mosquito vector as well as enhancing antibodies. We establish that SGE disrupts endothelial barrier function in the skin, induces vascular leak, and, in combination with enhancing antibodies, increases dendritic cell migration to skin-draining LNs. Because antibody-enhanced dengue pathogenesis occurs beyond the site of DENV-SGE co-inoculation in the skin, future studies are needed to determine whether SGE alters immune responses and thereby augments systemic dengue pathogenesis. Increasing DENV infection of cDCs and MΦs in the dermis could further modulate the priming of memory responses that determine pathogenesis during subsequent DENV infections. The role of mosquito-derived factors in dengue pathogenesis warrants further studies, and our findings call for additional research on arthropod-borne pathogens whose vectors share similar blood-feeding strategies and may also impair endothelial barrier function.

## Materials and Methods

### Ethics statement

Mice were bred and maintained in the University of California Berkeley Animal Facility, and experiments were performed strictly following guidelines of the American Veterinary Medical Association and the Guide for the Care and Use of Laboratory Animals of the National Institutes of Health. The University of California Berkeley’s Animal Care and Use Committee pre-approved all experiments (protocol AUP-2014-08-6638). Trained laboratory personnel performed anesthesia of mice via isoflurane inhalation and euthanasia of mice using exposure to CO_2_ followed by cervical dislocation.

### Mice

Wild-type C57BL/6 mice were purchased from the Jackson Laboratory. C57BL/6 mice deficient in the IFN-α/β receptor-1 (*Ifnar1* ^tm1Agt^, here termed *Ifnar* ^–/–^) were provided by Dr. Daniel Portnoy (University of California, Berkeley, CA) [82]. After DENV inoculation, mice were monitored following the standardized cumulative morbidity scale from 1 (healthy) to 5 (moribund) [28,31]: 1, healthy; 2, mild signs of lethargy; 3, fur ruffling, hunched posture; 4, increased lethargy, limited mobility; 5, moribund with minimal mobility and inability to reach food or water. Moribund mice were euthanized immediately, assigned a score of 5, and excluded from the mean morbidity calculations on subsequent days.

### Dengue virus

To mimic DENV transmission from an infected mosquito to the mammalian host, we inoculated *Ifnar* ^–/–^mice intradermally (i.d.) with DENV produced in C6/36 mosquito cells. We used the DENV2 strain D220, which was derived via 20 passages of the Taiwanese clinical isolate PL046 between C6/36 cells and serum of 129/Sv mice deficient in IFN-α/β and -*γ* receptors, acquiring defined mutations that increased its virulence in mice [29,31]. D220 stocks (hereafter termed DENV) were propagated in C6/36 cells (obtained from Dr. Paul Young, University of Queensland, Brisbane, Australia) at 28°C in M199 medium containing 10% fetal bovine serum (FBS), 100 U/ml penicillin/streptomycin, and GlutaMax (all from LifeTechnologies). T175 flasks containing 80% confluent C6/36 cells were infected with DENV at a multiplicity of infection of 0.05–0.1 in serum-free medium for 2 h and then supplemented with 2% FBS. Supernatants were harvested between days 5 and 8 post-infection and concentrated using Amicon Ultra-15 Centrifugal Filter Units with a 100 kDa molecular weight cut-off (Millipore) before storage at -80°C. DENV titers were determined via plaque assay using BHK-21 clone 15 cells, which were maintained at 37°C and 5% CO_2_ in α-MEM medium with 5% FBS, 100 U/ml penicillin/streptomycin, and GlutaMax. BHK-21 cells in 12-well plates (Becton Dickinson) at 60% confluence were infected with 150 μl DENV in 10-fold serial dilutions for 2 h before overlaying with 10% low-melting agarose (Cambrex) in fully supplemented α-MEM medium. BHK-21 cells were fixed 7 days later using 10% buffered formalin phosphate (Fisher Scientific) and stained with 2.5% crystal violet in 30% ethanol. DENV titers were calculated as plaque forming units (PFU)/ml.

### Mosquito salivary gland extract

Salivary gland extracts (SGE) were obtained from non-infected wild-caught Nicaraguan (DENV infections *in vivo*) or colonized Panamanian female *Ae*. *aegypti* mosquitoes (vascular permeability assays *in vitro* and *in vivo*). Mosquitoes were immobilized with CO_2_, dipped into 70% ethanol, and placed on glass slides in sterile PBS. The mosquito head was pulled away from the body, and the exposed salivary glands were dissected from the head or thorax under aseptic conditions and placed into fresh PBS (2 salivary glands/10 μl). Salivary gland membranes were disrupted via sonication in ice water at 100 mV for 3 bursts of 20 sec with 1 min of cooling time between bursts. Extracts were centrifuged at 5,000 g for 10 min at 4°C, and SGE was collected as supernatant. The protein concentration of the SGE stocks was 0.37–0.60 μg/ml, as estimated via NanoDrop (Thermo Scientific). SGE stocks had a neutral pH of 7.0 and were free of endotoxin or contained very low levels that were well below the FDA-approved limit for injection solutions of (<5 endotoxin units per kg per hour) as tested using the endpoint chromogenic Limulus Amebocyte Lysate kit (Lonza).

### Intradermal inoculation

Ears of anesthetized *Ifnar* ^–/–^mice were immobilized using cover slip forceps. A sterilized needle (30-gauge, 25-mm length, and 10°-12° bevel) used with a 25-μl reusable glass microinjection syringe (Hamilton) was inserted ~3 mm into the ventral side of the ear skin at a flat angle with the bevel pointing up, and DENV diluted in 20 μl PBS was slowly inoculated. To model the presence of mosquito-derived factors, we co-injected DENV mixed with the equivalent of 1 salivary gland from Nicaraguan mosquitoes in 20 μl PBS. Control ears were left untouched or were inoculated with SGE diluted in PBS or PBS alone. Naïve mice served to model 1° infections, whereas ADE conditions consisted of inoculation with a subneutralizing dose of 5 μg anti-DENV protein E monoclonal antibody 4G2 (ATCC) intraperitoneally in 200 μl PBS 24 h prior to DENV infection. In certain experiments, mice were anesthetized once again 4 h after i.d. DENV infection, and the inoculated site of the ear skin or an equivalent piece from the non-inoculated ear was surgically removed using sterile scissors. Bleeding was stopped immediately by applying pressure via sterile gauze, and mice were monitored closely for any direct adverse effects of the procedure. Only occasional scratching for less than 2 h was observed.

### Flow cytometric analysis

Organs of euthanized mice were collected on ice. Ears were incubated for 5 min at room temperature with hair removal cream (Nair), washed in PBS, and split into dorsal and ventral halves using tweezers (TDI). Skin samples were digested floating with the epidermal side up for 90 min with 2 U/ml Dispase II in HBSS without Ca^2+^/Mg^2+^ (LifeTech) in 5% CO_2_ at 37°C. The epidermis was then separated as one sheet from the dermis and each was placed into a tube containing RPMI 1640 medium with 10% FBS. Using scissors, the epidermal and dermal layers were cut into pieces of 1–2 mm size, PBS containing a final concentration of 1.6 mg/ml collagenase type-1 (LifeTech) and 10 U/ml DNase 1 (Roche) was added, and digestion of the epidermis dermis was allowed to proceed for 45 min and 80 min, respectively, at 37°C while shaking at 220 rotations per min. Homogeneous cell suspensions were generated via pipetting and filtering through 100 μm nylon meshes. Cervical LNs or spleens were crushed in medium under nylon meshes and digested in a final concentration of 1 mg/ml collagenase type-1 and 10 U/ml DNase 1 for 30 min at 37°C and 5% CO_2_ before generating single cell suspensions via pipetting. Spleen samples were treated with Red Blood Cell Lysis Buffer (eBioscience). Total numbers of live cells in LNs were determined via manual count and trypan blue exclusion.

Cells were stained with Zombie Aqua (BioLegend) in PBS to exclude dead cells. Cell surface markers were then stained in PBS without Ca^2+^/Mg^2+^ with 2% FBS and 2 mM EDTA (LifeTech) using the following monoclonal antibodies (from BioLegend or BD Pharmingen) to identify cell types: CD3 (17A2), CD4 (GK1.4), CD8 (53–67), CD11c (N418), CD11b (M1/70), CD44 (IM7), CD45 (30-F11), CD62L (MEL-14), CD103 (2E7), F4/80 (BM-8), Ly6C (AL21), Ly6G (1A8), MHCII (I-A/I-E, M5/114.15.2), CCR7 (4B12) or isotype control (RTK2758), and Fc*γ*R (2.4G2) or isotype control (A-95-1). The monoclonal antibodies were conjugated to PacificBlue, Brilliant Violet 605, PE, PE-CF594, PE-Cy7, Alexa Fluor 700, APC-Cy7, or biotin. Cells stained with biotinylated antibodies were visualized using PE-Cy7- or Brilliant Violet 605-conjugated streptavidin (BioLegend). Cells were fixed with 2% formaldehyde (Ted Pella) for 10 min at room temperature, and membranes were permeabilized with 0.1% saponin (Sigma) in PBS containing 2% FBS, 2 mM EDTA, and 1% normal mouse serum to block remaining fixative. To identify DENV-infected cells, cells were stained intracellularly with monoclonal antibodies directed against DENV non-structural protein NS3 (clone E1D8, [14]) and structural protein E (clone 4G2, ATCC) that were conjugated to Alexa Fluor 488 or Alexa Fluor 647, respectively, using protein-labeling kits (LifeTech). Intracellular staining for mannose receptor (C068C2) or isotype control (RTK2758) further dissected cell populations. Spleen cells were harvested 5.5 days post-i.d. DENV inoculation and were stained intracellularly for Ki-67 (SolA15) or isotype control (kit, eBiosciences) or after *in vitro* restimulation for 4 hours in RPMI 1640 medium with 10% FBS, 20 nM phorbol 12-myristate 13-acetate (Sigma), and 1 μM ionomycin (Sigma), with 5 μg/ml brefeldin A (eBiosciences) added during the final 2 hours were stained for IFN-*γ* (XMG1.2) or isotype control (RTK2071). Data were recorded using an LSR Fortessa cell analyzer (BD Biosciences) with 405, 488, 561, and 632 nm laser excitation lines and data analyzed using FlowJo 8.8.7 software (TreeStar). Gating FSC-A/SSC-A, SSC-H/SSC-W, FSC-H/FSC-W, and Zombie Aqua negative defined single, live-cell populations. The total number of cells in the dermis was determined via flow cytometer, while acquiring all cells for each sample and calculating total acquired cell counts.

### Quantification of DENV genomes

To determine DENV titers, a piece of less than 20 mg of tissue stored in RNAlater (Ambion) was homogenized for 1 min with 1-mm zirconia-silica beads using a Mini-Beadbeater-8 (BiospecProducts), and RNA was extracted after centrifugation using the RNeasy Mini Kit (Qiagen). RNA was extracted from 20 μl serum stored at -80°C using the QIAamp Viral RNA Mini Kit (Qiagen). RNA samples were eluted in a total volume of 50 μl, and 2 μl was used per qRT-PCR reaction in duplicate. DENV2 NS5 primer and probe sequences were as follows [83]: forward primer 5’-ACA AGT CGA ACA ACC TGG TCC AT, probe 5’-Fam TGG GAT TTC CTC CCA TGA TTC CAC TGG Tamra-Q, reverse primer 5’-GCC GCA CCA TTG GTC TTC TC (synthesized by Eurofins WMG Operon). DENV RNA and *Gapdh* mRNA (TaqMan Rodent *Gapdh* Control Reagents, ThermoFisher) were amplified in separate reactions using Verso one-step qRT-PCR Kits (ThermoFisher). A 7300 Real-Time PCR System (Applied Biosystems) was set to the following thermal cycler profile: reverse transcription at 50°C for 30 min, hot start at 95°C for 12.5 min, and for amplification and data acquisition, 40 PCR cycles consisting of denaturation at 95°C for 15 sec and annealing/extension at 60°C for 1 min.

### Endothelial permeability

Trans-endothelial electrical resistance (TEER) was used to measure endothelial permeability of confluent single-cell monolayers of human microvascular endothelial cells (HMEC-1) in Transwell Permeable Supports (Corning) using a voltohmmeter (World Precision Instruments) after adding 0.2, 0.5, 1 or 2 salivary gland equivalents to 300 μl medium in the apical or 500 μl in the basolateral compartment of the transwell insert. Adding 10 ng/ml of TNFα (R&D Systems) to HMEC-1 cells served as a positive control for increased endothelial permeability. After 24 h, 50% of the medium in the upper and lower chambers was replaced with fresh medium. Relative TEER was calculated as percent resistance in Ohms of the experimental condition divided by non-treated cells. To measure vascular permeability *in vivo*, wild-type mice were inoculated intravenously with 200 μg Dextran (10 kDa molecular weight) labeled with Alexa Fluor 680. After circulating for 5 min, one salivary gland equivalent from Panamanian mosquitoes was inoculated i.d. into mouse ears in 15 μl PBS. Control ears were inoculated with PBS alone or were left untouched. Mice were euthanized 30 min after i.d. inoculation, and ears were scanned using the Odyssey CLx Infrared Imaging System (Licor) to quantify fluorescence of a 3300 pixel size area. Fluorescence values of steady-state control ears were subtracted from values of ears inoculated with SGE or PBS.

### Statistical analysis

Numerical data were tested for statistically significant differences between experimental groups using unpaired parametric t-tests for flow cytometry data and DENV tissue viral load. A paired t-test was used to test for statistically significant differences between the fluorescence of the two ears of one animal injected with either PBS or SGE. Unpaired Mann-Whitney tests were used for non-parametric data, such as morbidity. Survival data were tested for statistically significant differences using the Log-rank (Mantel-Cox) test. Experimental groups were considered significantly different at *p*<0.05 and are marked on graphs as * for p<0.05, ** for p<0.01, *** for p<0.001, and **** for p<0.0001, or non-significant (n.s.). Data were plotted and statistically analyzed using Prism 6.0 software (GraphPad).

### Data availability

Data deposited in the Dryad repository: http://dx.doi.org/10.5061/dryad.4rd14 [84].

## Supporting Information

S1 Fig Related to Fig 1, SGE does not augment dengue pathogenesis after inoculation with 10^6^ PFU but augments pathogenesis after inoculation with 10^5^ PFU DENV in mice that were pre-exposed to SGE. (A-C) *Ifnar* ^–/–^mice were inoculated i.d. with 10^6^ PFU DENV in the absence (A) or presence (B) of enhancing antibodies. DENV was inoculated alone (as in [28]) or after mixing with *Ae*. *aegypti* SGE. (A-B) Bar graphs show mean morbidity ± SEM of mice on a scale from 1 = healthy to 5 = moribund. Statistically significant differences in morbidity between the presence and absence of SGE are marked on graphs as ** for p<0.01 and *** for p<0.001 or not significant (n.s.). The presence of enhancing antibodies significantly increased disease severity in the absence (day 4, p<0.01; days 5 and 6, p<0.001; day 7, p<0.01) and presence of SGE (day 3, p<0.01; days 4, 5, and 6, p<0.001). (C) Kaplan-Meier curves showing survival of mice. No significant differences in survival existed in the presence or absence of SGE. Significant differences in survival between the presence or absence of enhancing antibodies are marked as **, p<0.01. Data were pooled from three experiments, n = 8–9 mice per group. (D) *Ifnar* ^–/–^mice were pre-exposed i.d. three times with SGE or PBS at intervals of at least two weeks or were left untreated. Two weeks after the last injection, mice were infected i.d. in the presence of enhancing antibodies with 10^5^ PFU DENV mixed with SGE. SGE-naïve controls received DENV alone or DENV mixed with SGE as in Fig 1C. The Kaplan-Meier curves show survival of mice. Statistically significant differences are marked as * for p<0.1.
(TIF)

S2 Fig Related to Fig 2, gating and phenotype of immune cells in the dermis. *Ifnar* ^–/–^mice were inoculated i.d. with 10^5^ PFU DENV under 1° or ADE conditions in the presence or absence of SGE. (A) Contour plots showing live CD45^+^ hematopoietic cells in the dermis analyzed via flow cytometry in steady state or 14 h after inoculation. Starting at the second row from the top, CD11b^+^ cDCs were consecutively gated as MHCII^hi^ Langerin^-^CD11b^+^ Ly6C^-^, moDCs as MHCII^hi^ Langerin^-^CD11b^+^ Ly6C^+^, dermal Langerhans cells and CD103^+^ cDCs together as MHCII^+^ Langerin^+^, neutrophils as MHCII^low/–^Langerin^-^CD11b^hi^ Ly6G^+^, monocytes as MHCII^-^Langerin^-^CD11b^+^ Ly6G^-^ Ly6C^hi^, and MΦs as MHCII^low/–^Langerin^-^CD11b^hi^ Ly6G^-^ Ly6C^low/–^SSC^int-hi^. (B) Histogram overlays of surface stains (filled blue) for Fc*γ* receptors (CD16/32) or mannose receptor (CD206) or isotype controls (dashed black line) of cell populations gated as in (A) and lymphocytes gated as MHCII^-^CD11b^-^ Ly6G^-^ FSC^low^ SSC^low^ in the dermis of *Ifnar* ^–/–^mice that were infected with DENV in the presence of SGE and enhancing antibodies.
(TIF)

S3 Fig Related to Fig 3, frequency of cell populations in the dermis. (A-C) Bar graphs summarizing mean ± SEM percentage of neutrophils (A), monocytes (B), or moDCs (C) of CD45^+^ cells in the dermis of mice in steady state or 14 h after i.d. inoculation of 10^5^ PFU DENV in the presence or absence of SGE under 1° or ADE conditions. Populations were gated as in S2 Fig. Data were pooled from three experiments, n = 6–9 per group. Statistically significant differences between groups are marked as * for p<0.05, ** for p<0.01, and **** for p<0.0001.
(TIF)

S4 Fig Related to Fig 4, frequency of MigDCs, neutrophils, and monocytes as well as number of classical DCs in LNs. Mice were inoculated i.d. with PBS or 10^5^ PFU DENV under 1° or ADE conditions in the presence or absence of SGE, and skin-draining LNs were analyzed via flow cytometry after 14 h. (A) Histogram overlays of surface stains (filled blue) for chemokine receptor CCR7 or Fc*γ* receptors (CD16/32) or isotype controls (dashed black line) of cell populations in LNs, gated as in Fig 4. In addition, MHCII^-^lymphocytes that consisted mostly of T cells were gated as Ly6G^-^ CD11c^-^ CD11b^-^ F4/80^-^ MHCII^-^. (B-D) Bar graphs summarizing the percentage of MigDCs (B), neutrophils (C) or monocytes (D) of CD45^+^ cells in LNs. (E) Bar graph summarizing the number of cDCs in LNs. Data were pooled from three experiments, n = 6–9 per group. Statistically significant differences are marked as * for p<0.05, ** for p<0.01, *** for p<0.001, and **** for p<0.0001.
(TIF)

S5 Fig Related to Fig 4, T cell activation after i.d. DENV infection. Mice were left untreated or were inoculated i.d. with 10^5^ PFU DENV under ADE conditions in the presence or absence of SGE. Skin-draining LNs (A-B) or spleens (C-H) were analyzed via flow cytometry after 5.5 days. (A and C) Contour plots showing CD44 and CD62L expression of CD8^+^ CD3^+^ T cells. (B and D) Bar graphs summarizing percent CD44^+^ CD62L^+^ stimulated CD8^+^ or CD4^+^ CD3^+^ T cells in LNs (B) or spleen (D). (E) Contour plots showing CD44 expression and intracellular staining for Ki-67 of CD8^+^ T cells in the spleen. (F) Bar graphs summarizing percent Ki-67^+^ proliferating CD8^+^ or CD4^+^ T cells. (G) Contour plots showing CD8 expression and intracellular staining for IFN-*γ* of CD8^+^ T cells in the spleen after 4 h of *in vitro* restimulation. (H) Bar graphs summarizing percent IFN-*γ* expressing CD8^+^ or CD4^+^ T cells. Data were pooled from two experiments, n = 6 per group. Statistically significant differences are marked as * for p<0.05, ** for p<0.01, *** for p<0.001, and **** for p<0.0001.
(TIF)

S6 Fig Related to Fig 5, SGE disrupts endothelial barrier function when added to the basolateral or apical side of HMEC-1 monolayers. TEER of HMEC-1 monolayers grown in Transwell chambers was measured after adding the equivalent of 1 or 2 salivary glands or TNFα to basolateral (A-B) or apical compartments (C-D). TNFα was added as positive control for inducing endothelial permeability. (A and C) Graphs showing percent TEER relative to untreated control cells over time. (B and D) Bar graphs depicting percent TEER after 13 h of stimulation. Data were pooled from three experiments, n = 12 per group. Statistically significant differences are marked as **** for *p*<0.0001.
(TIF)

## References

[1] Ribeiro JM, Francischetti IM (2003) Role of arthropod saliva in blood feeding: sialome and post-sialome perspectives. Annu Rev Entomol 48: 73–88. 12194906 10.1146/annurev.ento.48.060402.102812

[2] Bhatt S, Gething PW, Brady OJ, Messina JP, Farlow AW, et al (2013) The global distribution and burden of dengue. Nature 496: 504–507. 10.1038/nature12060 23563266 PMC3651993

[3] Guzman MG, Alvarez M, Halstead SB (2013) Secondary infection as a risk factor for dengue hemorrhagic fever/dengue shock syndrome: an historical perspective and role of antibody-dependent enhancement of infection. Arch Virol 158: 1445–1459. 10.1007/s00705-013-1645-3 23471635

[4] Guzman MG, Harris E (2015) Dengue. Lancet 385: 453–465. 10.1016/S0140-6736(14)60572-9 25230594

[5] Vaughn DW, Green S, Kalayanarooj S, Innis BL, Nimmannitya S, et al (2000) Dengue viremia titer, antibody response pattern, and virus serotype correlate with disease severity. J Infect Dis 181: 2–9. 10608744 10.1086/315215

[6] Rothman AL (2011) Immunity to dengue virus: a tale of original antigenic sin and tropical cytokine storms. Nat Rev Immunol 11: 532–543. 10.1038/nri3014 21760609

[7] Halstead SB, O'Rourke EJ (1977) Antibody-enhanced dengue virus infection in primate leukocytes. Nature 265: 739–741. 404559 10.1038/265739a0

[8] Kou Z, Quinn M, Chen H, Rodrigo WW, Rose RC, et al (2008) Monocytes, but not T or B cells, are the principal target cells for dengue virus (DV) infection among human peripheral blood mononuclear cells. J Med Virol 80: 134–146. 18041019 10.1002/jmv.21051

[9] Blackley S, Kou Z, Chen H, Quinn M, Rose RC, et al (2007) Primary human splenic macrophages, but not T or B cells, are the principal target cells for dengue virus infection in vitro. J Virol 81: 13325–13334. 17928355 10.1128/JVI.01568-07 PMC2168870

[10] Schmid MA, Diamond MS, Harris E (2014) Dendritic cells in dengue virus infection: targets of virus replication and mediators of immunity. Front Immunol 5: 647 10.3389/fimmu.2014.00647 25566258 PMC4269190

[11] Durbin A, Vargas MJ, Thumar B, Hammond SN, Gordon G, et al (2008) Phenotyping of peripheral blood mononuclear cells during acute dengue illness demonstrates infection and increased activation of monocytes in severe cases compared to classic dengue fever. Virology 376: 429–435. 10.1016/j.virol.2008.03.028 18452966 PMC2546568

[12] Jessie K, Fong MY, Devi S, Lam SK, Wong KT (2004) Localization of dengue virus in naturally infected human tissues, by immunohistochemistry and in situ hybridization. J Infect Dis 189: 1411–1418. 15073678 10.1086/383043

[13] Kyle JL, Beatty PR, Harris E (2007) Dengue virus infects macrophages and dendritic cells in a mouse model of infection. J Infect Dis 195: 1808–1817. 17492597 10.1086/518007

[14] Balsitis SJ, Coloma J, Castro G, Alava A, Flores D, et al (2009) Tropism of dengue virus in mice and humans defined by viral nonstructural protein 3-specific immunostaining. Am J Trop Med Hyg 80: 416–424. 19270292

[15] Zellweger RM, Prestwood TR, Shresta S (2010) Enhanced infection of liver sinusoidal endothelial cells in a mouse model of antibody-induced severe dengue disease. Cell Host Microbe 7: 128–139. 10.1016/j.chom.2010.01.004 20153282 PMC2824513

[16] Prestwood TR, May MM, Plummer EM, Morar MM, Yauch LE, et al (2012) Trafficking and replication patterns reveal splenic macrophages as major targets of dengue virus in mice. J Virol 86: 12138–12147. 10.1128/JVI.00375-12 22933295 PMC3486461

[17] Briant L, Despres P, Choumet V, Misse D (2014) Role of skin immune cells on the host susceptibility to mosquito-borne viruses. Virology 464–465: 26–32. 10.1016/j.virol.2014.06.023 25043586

[18] Demeure CE, Brahimi K, Hacini F, Marchand F, Peronet R, et al (2005) Anopheles mosquito bites activate cutaneous mast cells leading to a local inflammatory response and lymph node hyperplasia. J Immunol 174: 3932–3940. 15778349 10.4049/jimmunol.174.7.3932

[19] Beghdadi W, Porcherie A, Schneider BS, Dubayle D, Peronet R, et al (2008) Inhibition of histamine-mediated signaling confers significant protection against severe malaria in mouse models of disease. J Exp Med 205: 395–408. 10.1084/jem.20071548 18227221 PMC2271011

[20] Titus RG, Ribeiro JM (1988) Salivary gland lysates from the sand fly Lutzomyia longipalpis enhance Leishmania infectivity. Science 239: 1306–1308. 3344436 10.1126/science.3344436

[21] Belkaid Y, Kamhawi S, Modi G, Valenzuela J, Noben-Trauth N, et al (1998) Development of a natural model of cutaneous leishmaniasis: powerful effects of vector saliva and saliva preexposure on the long-term outcome of Leishmania major infection in the mouse ear dermis. J Exp Med 188: 1941–1953. 9815271 10.1084/jem.188.10.1941 PMC2212417

[22] Styer LM, Lim PY, Louie KL, Albright RG, Kramer LD, et al (2011) Mosquito saliva causes enhancement of West Nile virus infection in mice. J Virol 85: 1517–1527. 10.1128/JVI.01112-10 21147918 PMC3028906

[23] Schneider BS, Soong L, Girard YA, Campbell G, Mason P, et al (2006) Potentiation of West Nile encephalitis by mosquito feeding. Viral Immunol 19: 74–82. 16553552 10.1089/vim.2006.19.74

[24] McCracken MK, Christofferson RC, Chisenhall DM, Mores CN (2014) Analysis of early dengue virus infection in mice as modulated by Aedes aegypti probing. J Virol 88: 1881–1889. 10.1128/JVI.01218-13 24198426 PMC3911562

[25] Cox J, Mota J, Sukupolvi-Petty S, Diamond MS, Rico-Hesse R (2012) Mosquito bite delivery of dengue virus enhances immunogenicity and pathogenesis in humanized mice. J Virol 86: 7637–7649. 10.1128/JVI.00534-12 22573866 PMC3416288

[26] Ader DB, Celluzzi C, Bisbing J, Gilmore L, Gunther V, et al (2004) Modulation of dengue virus infection of dendritic cells by Aedes aegypti saliva. Viral Immunol 17: 252–265. 15279703 10.1089/0882824041310496

[27] Cerny D, Haniffa M, Shin A, Bigliardi P, Tan BK, et al (2014) Selective susceptibility of human skin antigen presenting cells to productive dengue virus infection. PLoS Pathog 10: e1004548 10.1371/journal.ppat.1004548 25474532 PMC4256468

[28] Schmid MA, Harris E (2014) Monocyte recruitment to the dermis and differentiation to dendritic cells increases the targets for dengue virus replication. PLoS Pathog 10: e1004541 10.1371/journal.ppat.1004541 25474197 PMC4256458

[29] Shresta S, Sharar KL, Prigozhin DM, Beatty PR, Harris E (2006) A murine model for dengue lethal disease with increased vascular permeability. J Virol 80: 10208–10217. 17005698 10.1128/JVI.00062-06 PMC1617308

[30] Balsitis SJ, Williams KL, Lachica R, Flores D, Kyle JL, et al (2010) Lethal antibody enhancement of dengue disease in mice is prevented by Fc modification. PLoS Pathog 6: e1000790 10.1371/journal.ppat.1000790 20168989 PMC2820409

[31] Orozco S, Schmid MA, Parameswaran P, Lachica R, Henn MR, et al (2012) Characterization of a model of lethal dengue virus 2 infection in C57BL/6 mice deficient in the alpha/beta interferon receptor. J Gen Virol 93: 2152–2157. 10.1099/vir.0.045088-0 22815273 PMC3541791

[32] Yu CY, Chang TH, Liang JJ, Chiang RL, Lee YL, et al (2012) Dengue virus targets the adaptor protein MITA to subvert host innate immunity. PLoS Pathog 8: e1002780 10.1371/journal.ppat.1002780 22761576 PMC3386177

[33] Aguirre S, Maestre AM, Pagni S, Patel JR, Savage T, et al (2012) DENV inhibits type I IFN production in infected cells by cleaving human STING. PLoS Pathog 8: e1002934 10.1371/journal.ppat.1002934 23055924 PMC3464218

[34] Ashour J, Laurent-Rolle M, Shi PY, Garcia-Sastre A (2009) NS5 of dengue virus mediates STAT2 binding and degradation. J Virol 83: 5408–5418. 10.1128/JVI.02188-08 19279106 PMC2681973

[35] Shresta S, Kyle JL, Snider HM, Basanavapatna M, Beatty R, et al (2004) Interferon-dependent immunity is essential for resistance to primary dengue virus infection in mice, whereas T and B cell-dependent immunity is less critical. J Virol 78: 2701–2710. 14990690 10.1128/JVI.78.6.2701-2710.2004 PMC353772

[36] Ashour J, Morrison J, Laurent-Rolle M, Belicha-Villanueva A, Plumlee CR, et al (2010) Mouse STAT2 restricts early dengue virus replication. Cell Host Microbe 8: 410–421. 10.1016/j.chom.2010.10.007 21075352 PMC3310429

[37] Schneider BS, Soong L, Coffey LL, Stevenson HL, McGee CE, et al (2010) Aedes aegypti saliva alters leukocyte recruitment and cytokine signaling by antigen-presenting cells during West Nile virus infection. PLoS ONE 5: e11704 10.1371/journal.pone.0011704 20661470 PMC2908538

[38] Gomes R, Oliveira F (2012) The immune response to sand fly salivary proteins and its influence on leishmania immunity. Front Immunol 3: 110 10.3389/fimmu.2012.00110 22593758 PMC3349933

[39] Le Coupanec A, Babin D, Fiette L, Jouvion G, Ave P, et al (2013) Aedes mosquito saliva modulates Rift Valley fever virus pathogenicity. PLoS Negl Trop Dis 7: e2237 10.1371/journal.pntd.0002237 23785528 PMC3681724

[40] Conway MJ, Watson AM, Colpitts TM, Dragovic SM, Li Z, et al (2014) Mosquito saliva serine protease enhances dissemination of dengue virus into the mammalian host. J Virol 88: 164–175. 10.1128/JVI.02235-13 24131723 PMC3911723

[41] Styer LM, Kent KA, Albright RG, Bennett CJ, Kramer LD, et al (2007) Mosquitoes inoculate high doses of West Nile virus as they probe and feed on live hosts. PLoS Pathog 3: 1262–1270. 17941708 10.1371/journal.ppat.0030132 PMC1976553

[42] Boonnak K, Slike BM, Burgess TH, Mason RM, Wu SJ, et al (2008) Role of dendritic cells in antibody-dependent enhancement of dengue virus infection. J Virol 82: 3939–3951. 10.1128/JVI.02484-07 18272578 PMC2292981

[43] Miller JL, deWet BJ, Martinez-Pomares L, Radcliffe CM, Dwek RA, et al (2008) The mannose receptor mediates dengue virus infection of macrophages. PLoS Pathog 8: e17. 10.1371/journal.ppat.0040017 PMC2233670 18266465

[44] MartIn-Fontecha A, Sebastiani S, Hopken UE, Uguccioni M, Lipp M, et al (2003) Regulation of dendritic cell migration to the draining lymph node: impact on T lymphocyte traffic and priming. J Exp Med 198: 615–621. 12925677 10.1084/jem.20030448 PMC2194169

[45] Ohl L, Mohaupt M, Czeloth N, Hintzen G, Kiafard Z, et al (2004) CCR7 governs skin dendritic cell migration under inflammatory and steady-state conditions. Immunity 21: 279–288. 15308107 10.1016/j.immuni.2004.06.014

[46] Heath WR, Carbone FR (2013) The skin-resident and migratory immune system in steady state and memory: innate lymphocytes, dendritic cells and T cells. Nat Immunol 14: 978–985. 10.1038/ni.2680 24048119

[47] Merad M, Sathe P, Helft J, Miller J, Mortha A (2013) The dendritic cell lineage: ontogeny and function of dendritic cells and their subsets in the steady state and the inflamed setting. Annu Rev Immunol 31: 563–604. 10.1146/annurev-immunol-020711-074950 23516985 PMC3853342

[48] Amino R, Giovannini D, Thiberge S, Gueirard P, Boisson B, et al (2008) Host cell traversal is important for progression of the malaria parasite through the dermis to the liver. Cell Host Microbe 3: 88–96. 10.1016/j.chom.2007.12.007 18312843

[49] Turell MJ, Tammariello RF, Spielman A (1995) Nonvascular delivery of St. Louis encephalitis and Venezuelan equine encephalitis viruses by infected mosquitoes (Diptera: Culicidae) feeding on a vertebrate host. J Med Entomol 32: 563–568. 7650720 10.1093/jmedent/32.4.563

[50] Turell MJ, Spielman A (1992) Nonvascular delivery of Rift Valley fever virus by infected mosquitoes. Am J Trop Med Hyg 47: 190–194. 1503187 10.4269/ajtmh.1992.47.190

[51] Sidjanski S, Vanderberg JP (1997) Delayed migration of Plasmodium sporozoites from the mosquito bite site to the blood. Am J Trop Med Hyg 57: 426–429. 9347958 10.4269/ajtmh.1997.57.426

[52] Brown MG, Hermann LL, Issekutz AC, Marshall JS, Rowter D, et al (2011) Dengue virus infection of mast cells triggers endothelial cell activation. J Virol 85: 1145–1150. 10.1128/JVI.01630-10 21068256 PMC3019992

[53] Graham AC, Temple RM, Obar JJ (2015) Mast cells and influenza a virus: association with allergic responses and beyond. Front Immunol 6: 238 10.3389/fimmu.2015.00238 26042121 PMC4435071

[54] Luplertlop N, Misse D, Bray D, Deleuze V, Gonzalez JP, et al (2006) Dengue-virus-infected dendritic cells trigger vascular leakage through metalloproteinase overproduction. EMBO Rep 7: 1176–1181. 17028575 10.1038/sj.embor.7400814 PMC1679776

[55] Halstead SB, O'Rourke EJ (1977) Dengue viruses and mononuclear phagocytes. I. Infection enhancement by non-neutralizing antibody. J Exp Med 146: 201–217. 406347 10.1084/jem.146.1.201 PMC2180729

[56] Christofferson RC, McCracken MK, Johnson AM, Chisenhall DM, Mores CN (2013) Development of a transmission model for dengue virus. Virol J 10: 127 10.1186/1743-422X-10-127 23617898 PMC3659020

[57] Diamond MS, Roberts TG, Edgil D, Lu B, Ernst J, et al (2000) Modulation of dengue virus infection in human cells by alpha, beta, and gamma interferons. J Virol 74: 4957–4966. 10799569 10.1128/jvi.74.11.4957-4966.2000 PMC110847

[58] Tamoutounour S, Guilliams M, Montanana Sanchis F, Liu H, Terhorst D, et al (2013) Origins and functional specialization of macrophages and of conventional and monocyte-derived dendritic cells in mouse skin. Immunity 39: 925–938. 10.1016/j.immuni.2013.10.004 24184057

[59] Leon B, Lopez-Bravo M, Ardavin C (2007) Monocyte-derived dendritic cells formed at the infection site control the induction of protective T helper 1 responses against Leishmania. Immunity 26: 519–531. 17412618 10.1016/j.immuni.2007.01.017

[60] Ng LG, Qin JS, Roediger B, Wang Y, Jain R, et al (2011) Visualizing the neutrophil response to sterile tissue injury in mouse dermis reveals a three-phase cascade of events. J Invest Dermatol 131: 2058–2068. 10.1038/jid.2011.179 21697893

[61] Peters NC, Egen JG, Secundino N, Debrabant A, Kimblin N, et al (2008) In vivo imaging reveals an essential role for neutrophils in leishmaniasis transmitted by sand flies. Science 321: 970–974. 10.1126/science.1159194 18703742 PMC2606057

[62] Peters NC, Kimblin N, Secundino N, Kamhawi S, Lawyer P, et al (2009) Vector transmission of leishmania abrogates vaccine-induced protective immunity. PLoS Pathog 5: e1000484 10.1371/journal.ppat.1000484 19543375 PMC2691580

[63] Juffrie M, van Der Meer GM, Hack CE, Haasnoot K, Sutaryo, et al (2000) Inflammatory mediators in dengue virus infection in children: interleukin-8 and its relationship to neutrophil degranulation. Infect Immun 68: 702–707. 10639436 10.1128/iai.68.2.702-707.2000 PMC97195

[64] Taweechaisupapong S, Sriurairatana S, Angsubhakorn S, Yoksan S, Khin MM, et al (1996) Langerhans cell density and serological changes following intradermal immunisation of mice with dengue 2 virus. J Med Microbiol 45: 138–145. 8683550 10.1099/00222615-45-2-138

[65] Limon-Flores AY, Perez-Tapia M, Estrada-Garcia I, Vaughan G, Escobar-Gutierrez A, et al (2005) Dengue virus inoculation to human skin explants: an effective approach to assess in situ the early infection and the effects on cutaneous dendritic cells. Int J Exp Pathol 86: 323–334. 16191104 10.1111/j.0959-9673.2005.00445.x PMC2517443

[66] Zeidner NS, Higgs S, Happ CM, Beaty BJ, Miller BR (1999) Mosquito feeding modulates Th1 and Th2 cytokines in flavivirus susceptible mice: an effect mimicked by injection of sialokinins, but not demonstrated in flavivirus resistant mice. Parasite Immunol 21: 35–44. 10081770 10.1046/j.1365-3024.1999.00199.x

[67] Wanasen N, Nussenzveig RH, Champagne DE, Soong L, Higgs S (2004) Differential modulation of murine host immune response by salivary gland extracts from the mosquitoes Aedes aegypti and Culex quinquefasciatus. Med Vet Entomol 18: 191–199. 15189245 10.1111/j.1365-2915.2004.00498.x

[68] Cross ML, Cupp EW, Enriquez FJ (1994) Differential modulation of murine cellular immune responses by salivary gland extract of Aedes aegypti. Am J Trop Med Hyg 51: 690–696. 7985763 10.4269/ajtmh.1994.51.690

[69] Schneider BS, Soong L, Zeidner NS, Higgs S (2004) Aedes aegypti salivary gland extracts modulate anti-viral and TH1/TH2 cytokine responses to sindbis virus infection. Viral Immunol 17: 565–573. 15671753 10.1089/vim.2004.17.565

[70] Donovan MJ, Messmore AS, Scrafford DA, Sacks DL, Kamhawi S, et al (2007) Uninfected mosquito bites confer protection against infection with malaria parasites. Infect Immun 75: 2523–2530. 17339356 10.1128/IAI.01928-06 PMC1865743

[71] Kamhawi S, Belkaid Y, Modi G, Rowton E, Sacks D (2000) Protection against cutaneous leishmaniasis resulting from bites of uninfected sand flies. Science 290: 1351–1354. 11082061 10.1126/science.290.5495.1351

[72] Valenzuela JG, Belkaid Y, Garfield MK, Mendez S, Kamhawi S, et al (2001) Toward a defined anti-Leishmania vaccine targeting vector antigens: characterization of a protective salivary protein. J Exp Med 194: 331–342. 11489952 10.1084/jem.194.3.331 PMC2193460

[73] Thiakaki M, Rohousova I, Volfova V, Volf P, Chang KP, et al (2005) Sand fly specificity of saliva-mediated protective immunity in Leishmania amazonensis-BALB/c mouse model. Microbes Infect 7: 760–766. 15866511 10.1016/j.micinf.2005.01.013

[74] Oliveira F, Rowton E, Aslan H, Gomes R, Castrovinci PA, et al (2015) A sand fly salivary protein vaccine shows efficacy against vector-transmitted cutaneous leishmaniasis in nonhuman primates. Sci Transl Med 7: 290ra290. 10.1126/scitranslmed.aaa3043 26041707

[75] Oliveira F, Lawyer PG, Kamhawi S, Valenzuela JG (2008) Immunity to distinct sand fly salivary proteins primes the anti-Leishmania immune response towards protection or exacerbation of disease. PLoS Negl Trop Dis 2: e226 10.1371/journal.pntd.0000226 18414648 PMC2291569

[76] Schneider BS, McGee CE, Jordan JM, Stevenson HL, Soong L, et al (2007) Prior exposure to uninfected mosquitoes enhances mortality in naturally-transmitted West Nile virus infection. PLoS ONE 2: e1171 18000543 10.1371/journal.pone.0001171 PMC2048662

[77] Reagan KL, Machain-Williams C, Wang T, Blair CD (2012) Immunization of mice with recombinant mosquito salivary protein D7 enhances mortality from subsequent West Nile virus infection via mosquito bite. PLoS Negl Trop Dis 6: e1935 10.1371/journal.pntd.0001935 23236530 PMC3516580

[78] Machain-Williams C, Mammen MP Jr., Zeidner NS, Beaty BJ, Prenni JE, et al (2012) Association of human immune response to Aedes aegypti salivary proteins with dengue disease severity. Parasite Immunol 34: 15–22. 10.1111/j.1365-3024.2011.01339.x 21995849 PMC3240707

[79] Moser LA, Lim PY, Styer LM, Kramer LD, Bernard KA (2015) Parameters of Mosquito-Enhanced West Nile Virus Infection. J Virol 90: 292–299. 10.1128/JVI.02280-15 26468544 PMC4702546

[80] Marinotti O, James AA, Ribeiro J (1990) Diet and salivation in female Aedes aegypti mosquitoes. J Insect Physiol 36: 545–548.

[81] Wasserman HA, Singh S, Champagne DE (2004) Saliva of the Yellow Fever mosquito, Aedes aegypti, modulates murine lymphocyte function. Parasite Immunol 26: 295–306. 15541033 10.1111/j.0141-9838.2004.00712.x

[82] Muller U, Steinhoff U, Reis LF, Hemmi S, Pavlovic J, et al (1994) Functional role of type I and type II interferons in antiviral defense. Science 264: 1918–1921. 8009221 10.1126/science.8009221

[83] Laue T, Emmerich P, Schmitz H (1999) Detection of dengue virus RNA in patients after primary or secondary dengue infection by using the TaqMan automated amplification system. J Clin Microbiol 37: 2543–2547. 10405398 10.1128/jcm.37.8.2543-2547.1999 PMC85278

[84] Schmid MA, Glasner DR, Shah S, Michlmayr D, Kramer LD, and Harris E (2016). Data from: Mosquito saliva increases endothelial permeability in the skin, immune cell migration, and dengue pathogenesis during antibody-dependent enhancement. Dryad Digital Repository. 10.5061/dryad.4rd14. PMC4911004 27310141

