# Supplementary figures and images for: Mosquito Saliva Increases Endothelial Permeability in the Skin, Immune Cell Migration, and Dengue Pathogenesis during Antibody-Dependent Enhancement
Source: PLoS Pathog. 2016 Jun 16;12(6):e1005676. doi: 10.1371/journal.ppat.1005676 (PMC4911004; doi:10.1371/journal.ppat.1005676)

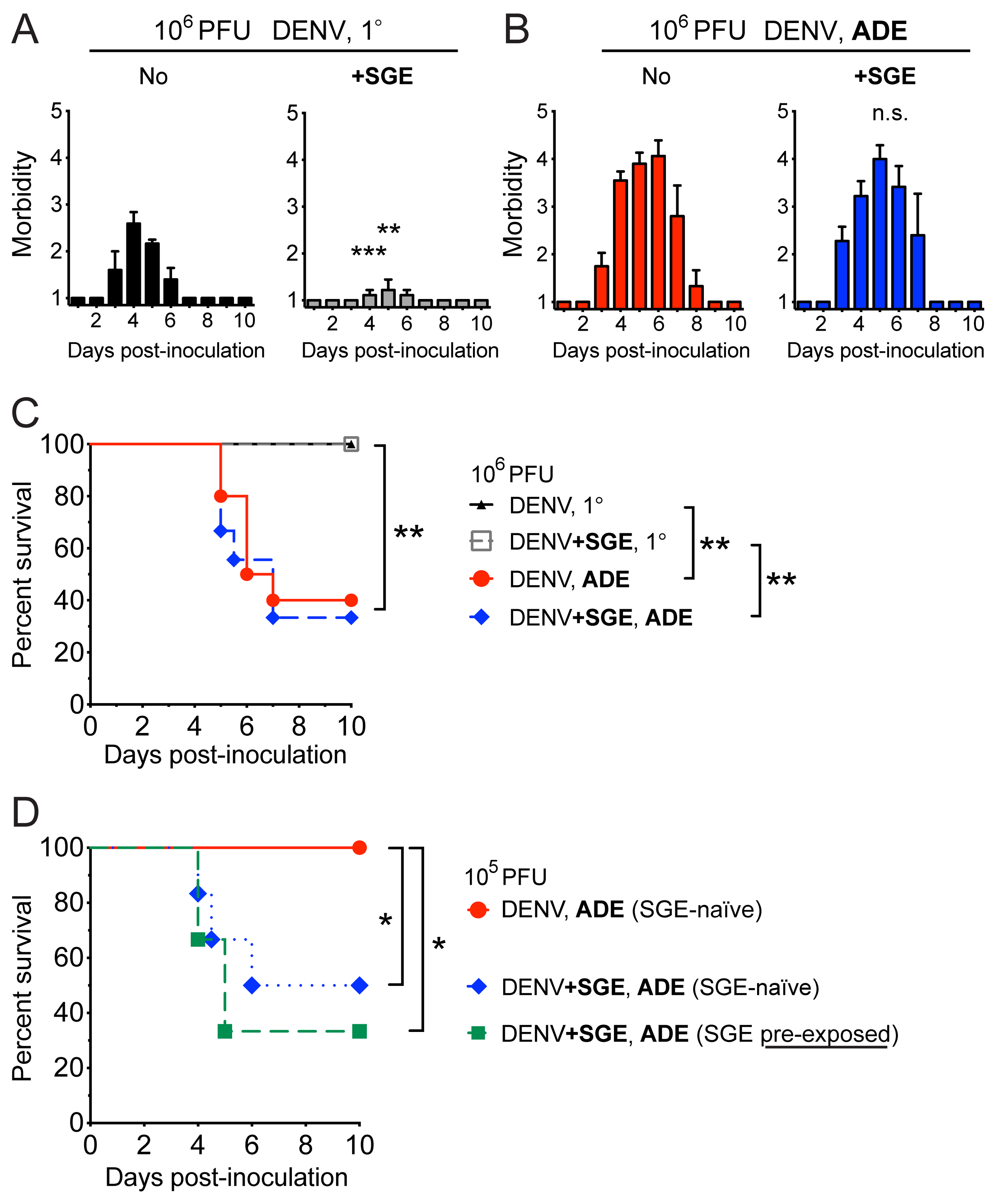

Supplement: S1 Fig — (A-C) Ifnar –/–mice were inoculated i.d. with 106 PFU DENV in the absence (A) or presence (B) of enhancing antibodies. DENV was inoculated alone (as in [28]) or after mixing with Ae. aegypti SGE. (A-B) Bar graphs show mean morbidity ± SEM of mice on a scale from 1 = healthy to 5 = moribund. Statistically significant differences in morbidity between the presence and absence of SGE are marked on graphs as ** for p<0.01 and *** for p<0.001 or not significant (n.s.). The presence of enhancing antibodies significantly increased disease severity in the absence (day 4, p<0.01; days 5 and 6, p<0.001; day 7, p<0.01) and presence of SGE (day 3, p<0.01; days 4, 5, and 6, p<0.001). (C) Kaplan-Meier curves showing survival of mice. No significant differences in survival existed in the presence or absence of SGE. Significant differences in survival between the presence or absence of enhancing antibodies are marked as **, p<0.01. Data were pooled from three experiments, n = 8–9 mice per group. (D) Ifnar –/–mice were pre-exposed i.d. three times with SGE or PBS at intervals of at least two weeks or were left untreated. Two weeks after the last injection, mice were infected i.d. in the presence of enhancing antibodies with 105 PFU DENV mixed with SGE. SGE-naïve controls received DENV alone or DENV mixed with SGE as in Fig 1C. The Kaplan-Meier curves show survival of mice. Statistically significant differences are marked as * for p<0.1. (TIF) [file ppat.1005676.s001.tif]

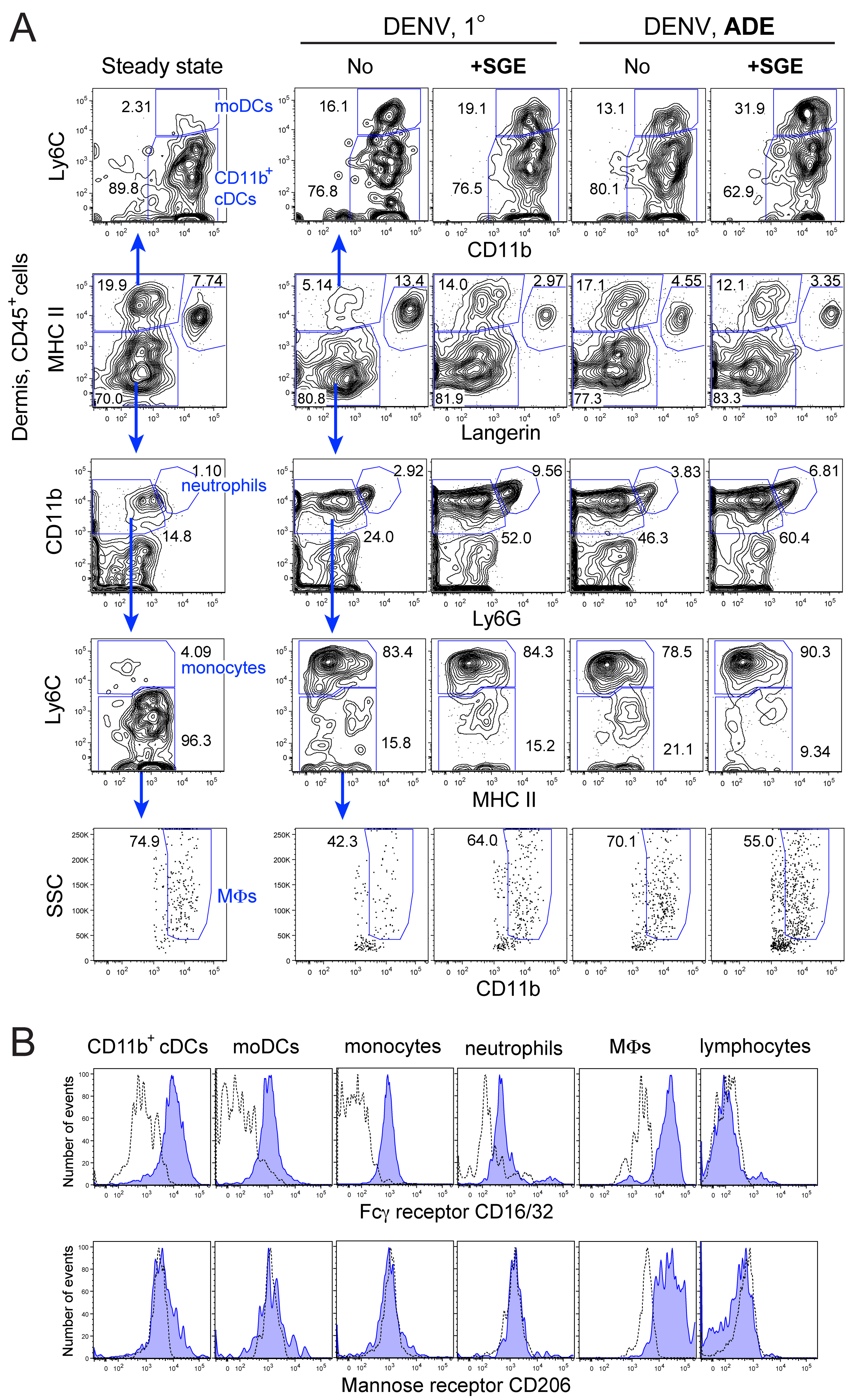

Supplement: S2 Fig — Ifnar –/–mice were inoculated i.d. with 105 PFU DENV under 1° or ADE conditions in the presence or absence of SGE. (A) Contour plots showing live CD45+ hematopoietic cells in the dermis analyzed via flow cytometry in steady state or 14 h after inoculation. Starting at the second row from the top, CD11b+ cDCs were consecutively gated as MHCIIhi Langerin-CD11b+ Ly6C-, moDCs as MHCIIhi Langerin-CD11b+ Ly6C+, dermal Langerhans cells and CD103+ cDCs together as MHCII+ Langerin+, neutrophils as MHCIIlow/–Langerin-CD11bhi Ly6G+, monocytes as MHCII-Langerin-CD11b+ Ly6G- Ly6Chi, and MΦs as MHCIIlow/–Langerin-CD11bhi Ly6G- Ly6Clow/–SSCint-hi. (B) Histogram overlays of surface stains (filled blue) for Fcγ receptors (CD16/32) or mannose receptor (CD206) or isotype controls (dashed black line) of cell populations gated as in (A) and lymphocytes gated as MHCII-CD11b- Ly6G- FSClow SSClow in the dermis of Ifnar –/–mice that were infected with DENV in the presence of SGE and enhancing antibodies. (TIF) [file ppat.1005676.s002.tif]

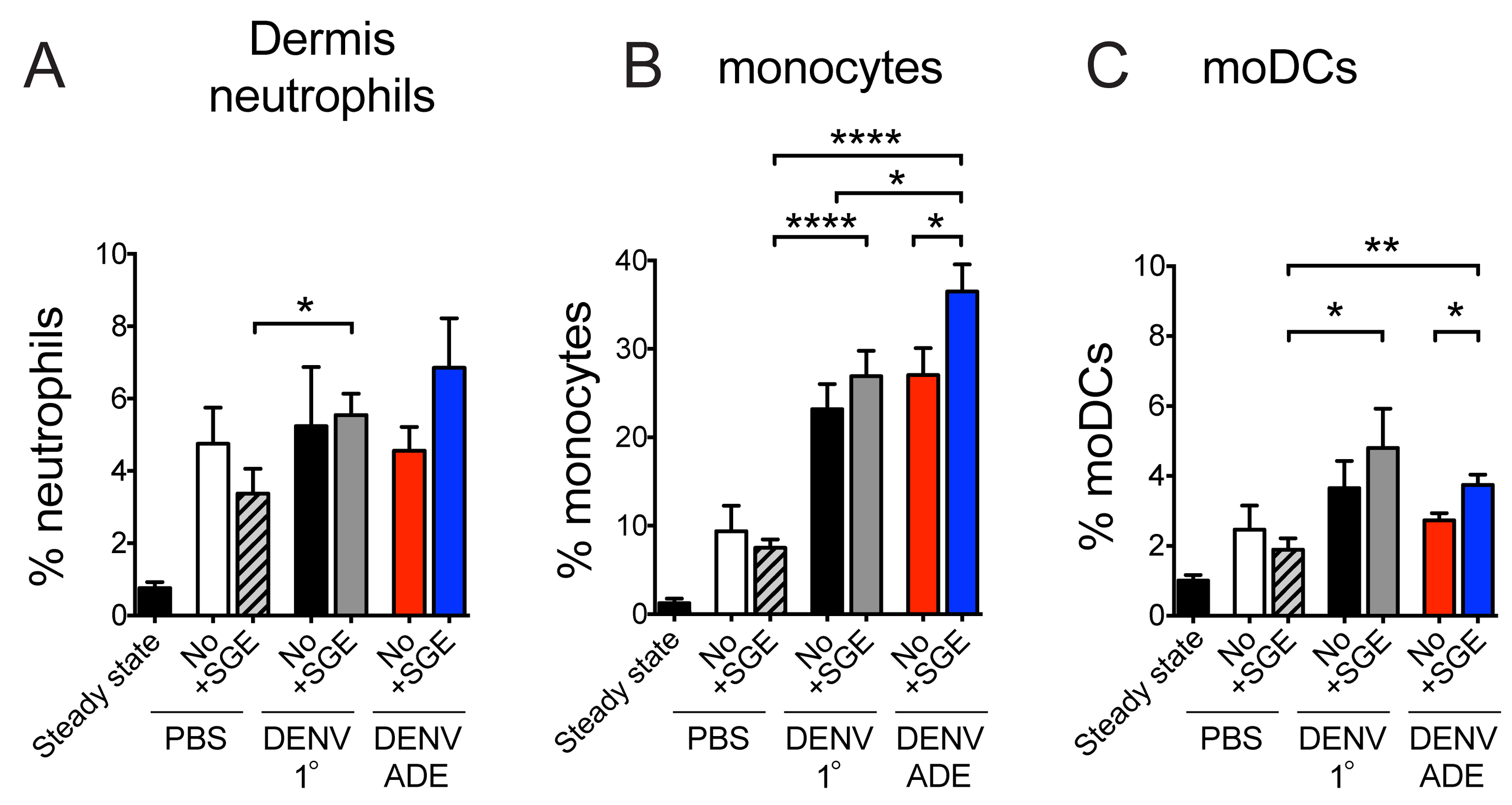

Supplement: S3 Fig — (A-C) Bar graphs summarizing mean ± SEM percentage of neutrophils (A), monocytes (B), or moDCs (C) of CD45+ cells in the dermis of mice in steady state or 14 h after i.d. inoculation of 105 PFU DENV in the presence or absence of SGE under 1° or ADE conditions. Populations were gated as in S2 Fig. Data were pooled from three experiments, n = 6–9 per group. Statistically significant differences between groups are marked as * for p<0.05, ** for p<0.01, and **** for p<0.0001. (TIF) [file ppat.1005676.s003.tif]

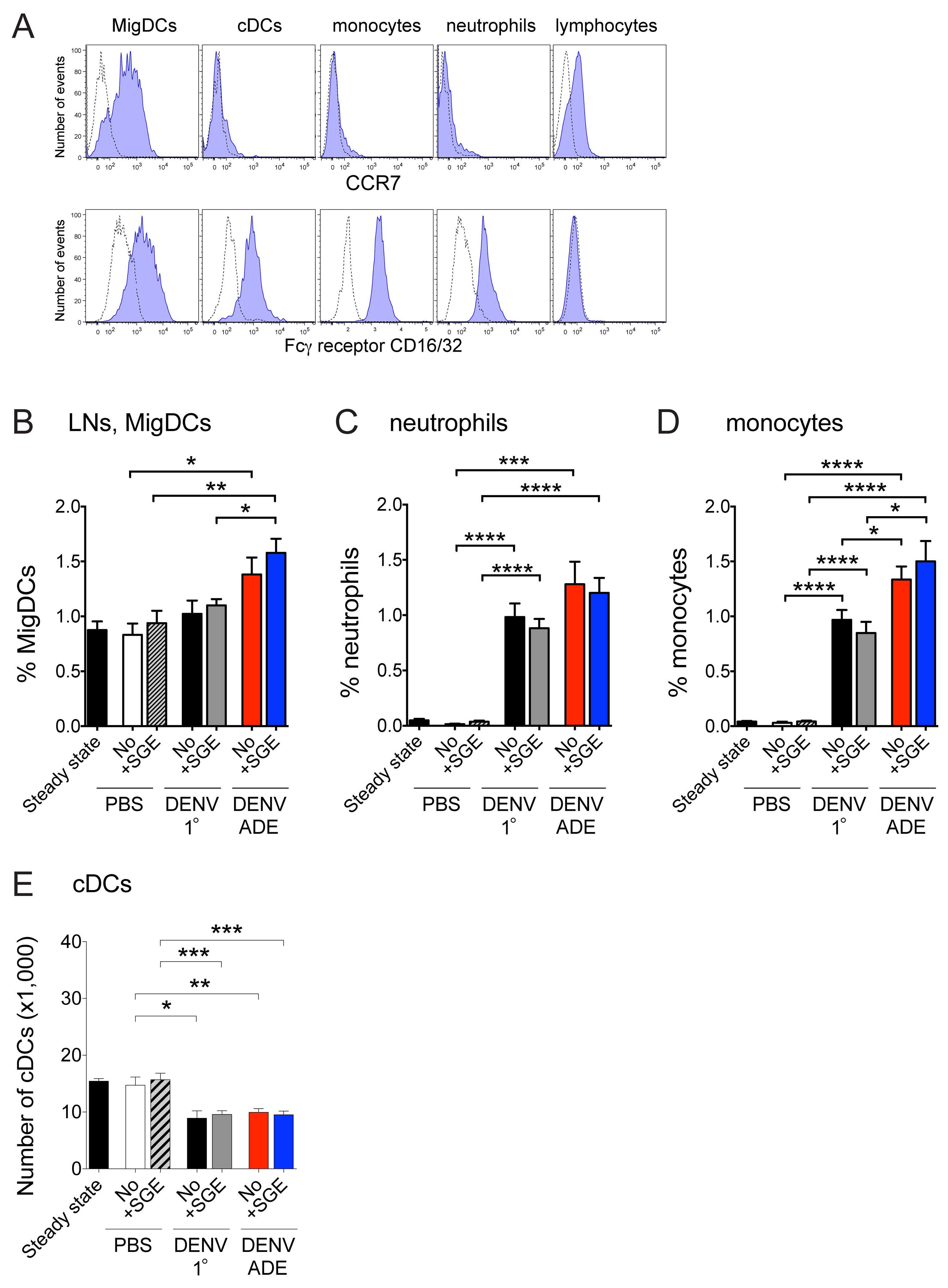

Supplement: S4 Fig — Mice were inoculated i.d. with PBS or 105 PFU DENV under 1° or ADE conditions in the presence or absence of SGE, and skin-draining LNs were analyzed via flow cytometry after 14 h. (A) Histogram overlays of surface stains (filled blue) for chemokine receptor CCR7 or Fcγ receptors (CD16/32) or isotype controls (dashed black line) of cell populations in LNs, gated as in Fig 4. In addition, MHCII-lymphocytes that consisted mostly of T cells were gated as Ly6G- CD11c- CD11b- F4/80- MHCII-. (B-D) Bar graphs summarizing the percentage of MigDCs (B), neutrophils (C) or monocytes (D) of CD45+ cells in LNs. (E) Bar graph summarizing the number of cDCs in LNs. Data were pooled from three experiments, n = 6–9 per group. Statistically significant differences are marked as * for p<0.05, ** for p<0.01, *** for p<0.001, and **** for p<0.0001. (TIF) [file ppat.1005676.s004.tif]

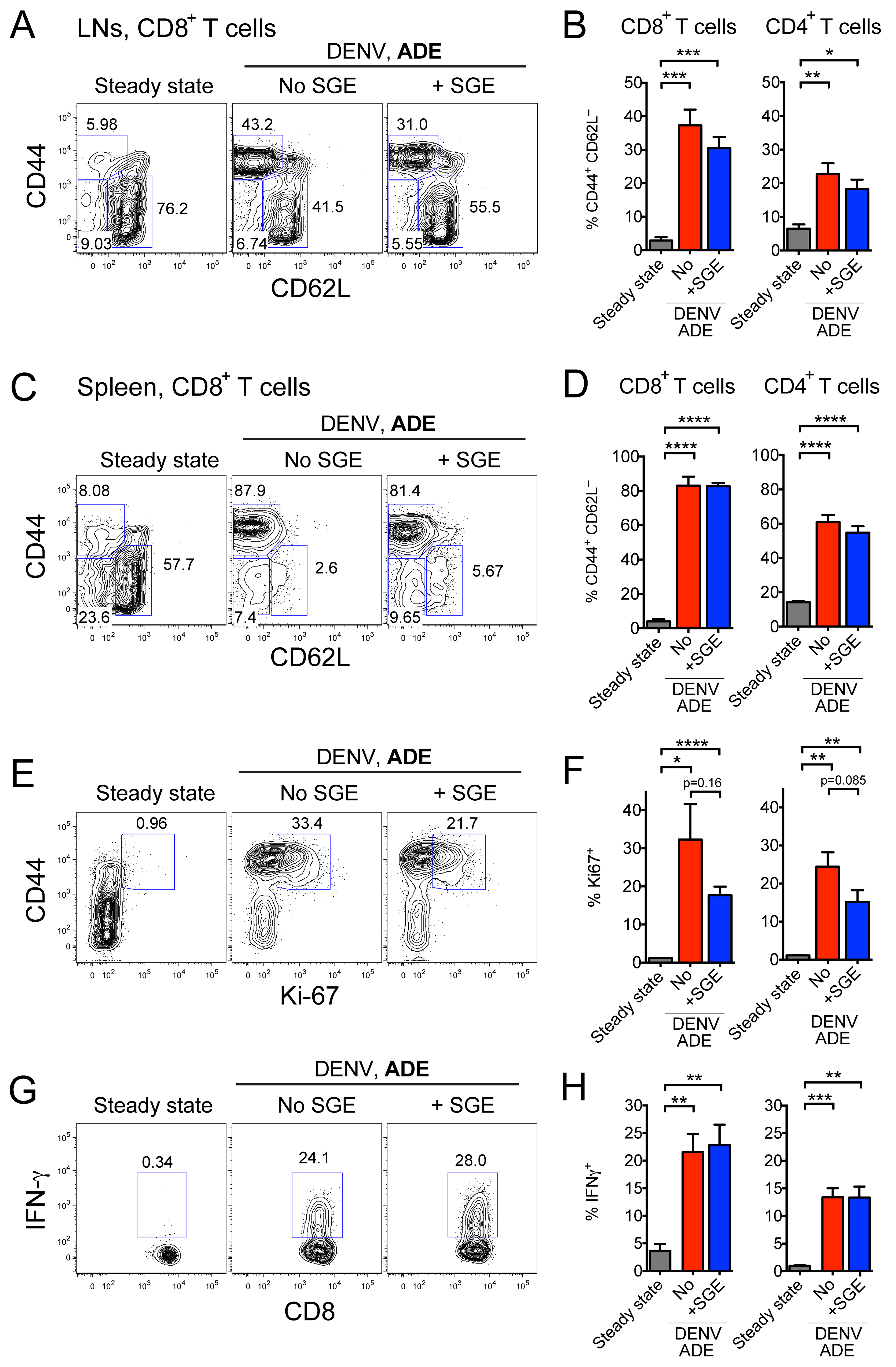

Supplement: S5 Fig — Mice were left untreated or were inoculated i.d. with 105 PFU DENV under ADE conditions in the presence or absence of SGE. Skin-draining LNs (A-B) or spleens (C-H) were analyzed via flow cytometry after 5.5 days. (A and C) Contour plots showing CD44 and CD62L expression of CD8+ CD3+ T cells. (B and D) Bar graphs summarizing percent CD44+ CD62L+ stimulated CD8+ or CD4+ CD3+ T cells in LNs (B) or spleen (D). (E) Contour plots showing CD44 expression and intracellular staining for Ki-67 of CD8+ T cells in the spleen. (F) Bar graphs summarizing percent Ki-67+ proliferating CD8+ or CD4+ T cells. (G) Contour plots showing CD8 expression and intracellular staining for IFN-γ of CD8+ T cells in the spleen after 4 h of in vitro restimulation. (H) Bar graphs summarizing percent IFN-γ expressing CD8+ or CD4+ T cells. Data were pooled from two experiments, n = 6 per group. Statistically significant differences are marked as * for p<0.05, ** for p<0.01, *** for p<0.001, and **** for p<0.0001. (TIF) [file ppat.1005676.s005.tif]

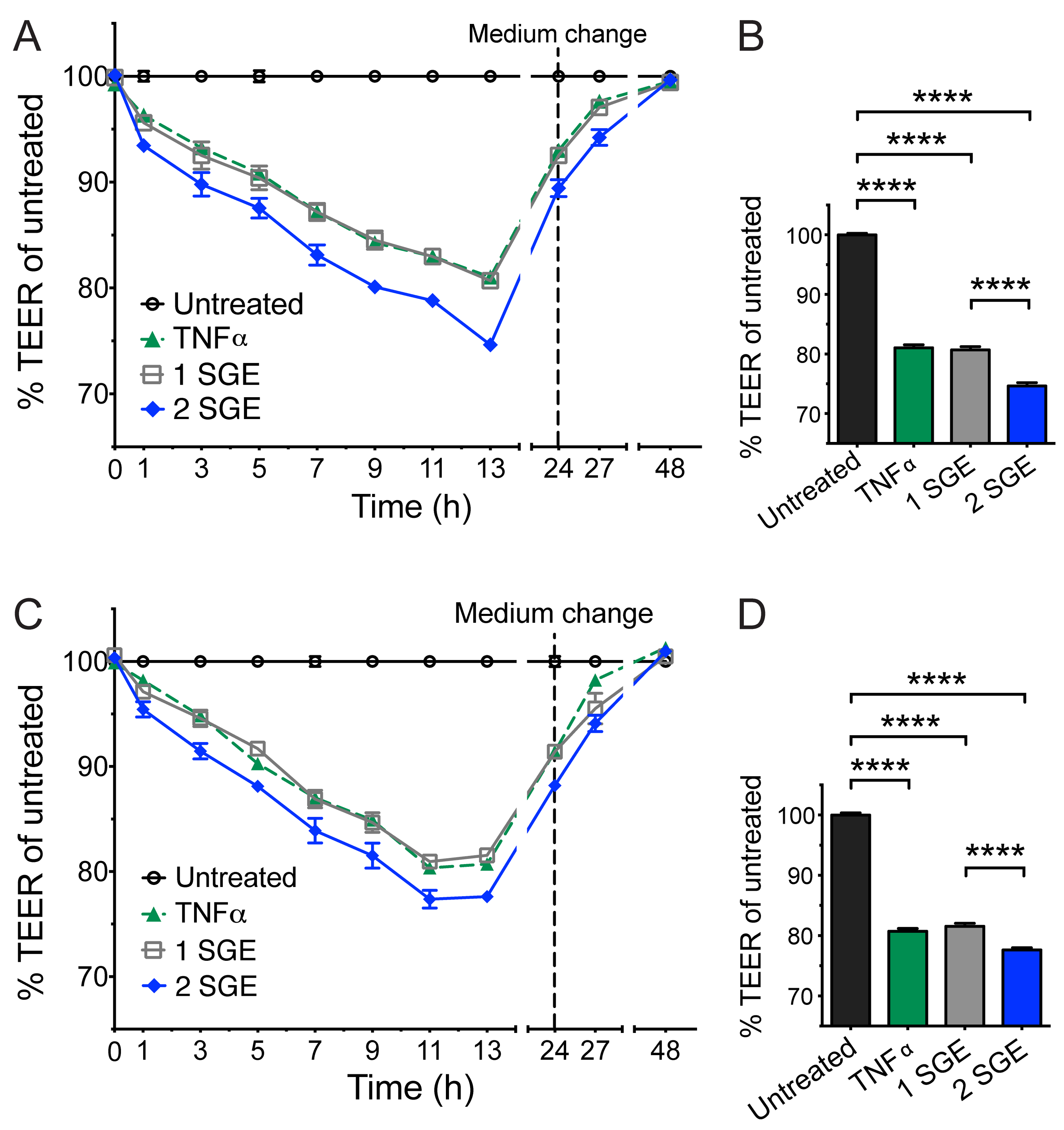

Supplement: S6 Fig — TEER of HMEC-1 monolayers grown in Transwell chambers was measured after adding the equivalent of 1 or 2 salivary glands or TNFα to basolateral (A-B) or apical compartments (C-D). TNFα was added as positive control for inducing endothelial permeability. (A and C) Graphs showing percent TEER relative to untreated control cells over time. (B and D) Bar graphs depicting percent TEER after 13 h of stimulation. Data were pooled from three experiments, n = 12 per group. Statistically significant differences are marked as **** for p<0.0001. (TIF) [file ppat.1005676.s006.tif]
